# Precision Oncology via Radiotherapy‐Triggered Drug Delivery: Mechanisms, Molecular Engineering, and Clinical Translation

**DOI:** 10.1002/advs.77450

**Published:** 2026-08-25

**Authors:** Jianv Jiang, Tanfeng Zhang, Junliang Dong, Wenjing Sun

**Affiliations:** ^1^ Key Lab of Artificial Organs and Computational Medicine Institute of Translational Medicine Shulan International Medical College, Zhejiang Shuren University Hangzhou Zhejiang People's Republic of China; ^2^ Department of Cardiology Sir Run Run Shaw Hospital Zhejiang University School of Medicine Hangzhou People's Republic of China; ^3^ Zhejiang Key Laboratory of Cardiovascular Intervention and Precision Medicine Hangzhou People's Republic of China; ^4^ Engineering Research Center for Cardiovascular Innovative Devices of Zhejiang Province Hangzhou People's Republic of China

**Keywords:** dosimetry‐governed activation, molecular actuation, radiation‐responsive nanoplatforms, radiochemical mechanisms, radiotherapy‐triggered drug delivery systems (RDDS)

## Abstract

Radiotherapy‐triggered drug delivery systems (RDDS) promise to integrate the spatial precision of ionizing radiation with controllable pharmacological activation. However, clinical translation remains constrained by its reliance on supra‐clinical irradiation doses. Here, we present a unifying framework redefining RDDS through two distinct paradigms: structural disassembly and molecular actuation. We delineate their radiochemical foundations, highlighting reductive and electron‐driven mechanisms as more robust and tumor‐selective than stochastic reactive oxygen species‐mediated pathways. Furthermore, we position radiation‐driven gas therapy as a distinct modality based on in situ molecular generation. Critically, we identify the dose–response mismatch between radiolytic chemistry and clinical radiation as the central translational bottleneck. To overcome this, we propose a design paradigm centered on highly predictable, dose‐matched activation, where mechanisms directly coupling radiation energy to molecular transformation offer superior predictability. Finally, we explore the integration of RDDS with imaging and artificial intelligence to create closed‐loop, feedback‐controlled systems. This review establishes a conceptual and translational roadmap, envisioning the evolution of radiotherapy from a purely cytotoxic modality into a programmable actuator for precision oncology.

## Graphical abstract

This review redefines radiotherapy‐triggered drug delivery systems through structural disassembly and molecular actuation, proposing a translation‐oriented framework for dose‐correlated activation to advance clinically integrated precision oncology.

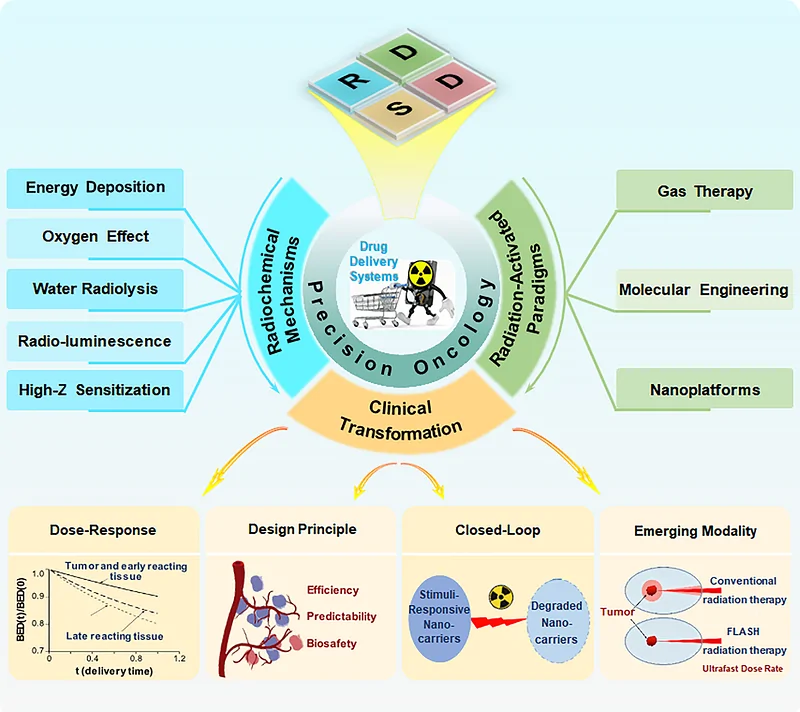

## 1 Introduction

Radiotherapy (RT) remains a cornerstone of modern oncology, employed in approximately half of all cancer patients due to its unparalleled ability to deliver spatially precise energy deposition deep within tissues [1]. By inducing DNA double‐strand breaks through ionizing radiation such as X‐rays, γ‐rays, electrons, and protons, RT achieves effective local tumor control [2]. However, its clinical efficacy is fundamentally constrained by two persistent limitations: the radio‐resistance of hypoxic tumor regions and dose‐limiting toxicity to surrounding normal tissues [3]. In parallel, systemic chemotherapy, while essential for controlling disseminated disease, suffers from non‐specific biodistribution, resulting in severe off‐target toxicities that often necessitate dose reduction or treatment discontinuation [4]. Concurrent chemoradiotherapy partially addresses these challenges by leveraging radio‐sensitization, yet it frequently introduces compounded toxicity without resolving the underlying issue of spatiotemporal drug control [5].

The emergence of nanomedicine initially offered a potential solution through the enhanced permeability and retention (EPR) effect, enabling preferential accumulation of drug carriers in tumor tissues [6]. However, clinical translation has been limited by physiological barriers, heterogeneous tumor uptake, and the lack of precise control over drug release [7]. These limitations have driven the development of stimuli‐responsive drug delivery systems, in which specific internal or external cues trigger therapeutic activation [8, 9, 10, 11]. While endogenous stimuli—such as pH, enzymatic activity, or redox gradients—are inherently variable and patient‐dependent, exogenous triggers provide superior controllability [12, 13]. Among these, ionizing radiation is uniquely positioned due to its virtually unlimited tissue penetration and its integration within highly standardized clinical workflows, including intensity‐modulated radiotherapy (IMRT) and stereotactic body radiotherapy (SBRT), making it an ideal external trigger for spatiotemporally controlled drug activation [14, 15, 16].

Against this backdrop, radiotherapy‐triggered drug delivery systems (RDDS) have emerged as a promising strategy to couple the physical precision of RT with on‐demand pharmacological activation [17, 18, 19, 20]. Over the past decade, a wide range of RDDS platforms have been developed, spanning small‐molecule prodrugs, polymeric nanocarriers, inorganic–organic hybrids, and gas‐releasing systems [21, 22, 23]. Despite this diversity, the field remains conceptually fragmented, with distinct material systems and activation mechanisms often treated in isolation. More critically, a fundamental translational bottleneck persists: the mismatch between the radiation dose required to activate chemical or nanostructured systems and the dose that can be safely delivered in clinical practice [24]. At clinically relevant doses (typically 1.8–2.2 Gy per fraction), the yield of radiolytic species is intrinsically low, limiting the efficiency of many radiation‐responsive processes [25]. As a result, numerous RDDS platforms demonstrate robust activation under high‐dose experimental conditions but fail to achieve meaningful performance under clinically realistic irradiation, establishing the dose–response gap as a central challenge that cannot be resolved by incremental material optimization alone.

In parallel, an equally critical but less explicitly recognized limitation lies in the lack of predictability in many RDDS platforms [26]. In modern radiotherapy, absorbed dose is a precisely quantified and spatially controlled parameter; however, in many existing systems—particularly those relying on reactive oxygen species (ROS)—drug activation depends on stochastic processes influenced by radical diffusion, recombination, oxygen availability, and intracellular antioxidant levels [27]. This results in substantial variability in therapeutic output even under identical irradiation conditions, undermining reproducibility and complicating clinical translation [28]. These considerations highlight a crucial shift in design philosophy: responsiveness alone is insufficient; instead, RDDS must establish a quantitative and reproducible relationship between radiation dose and chemical transformation.

**FIGURE 1:**
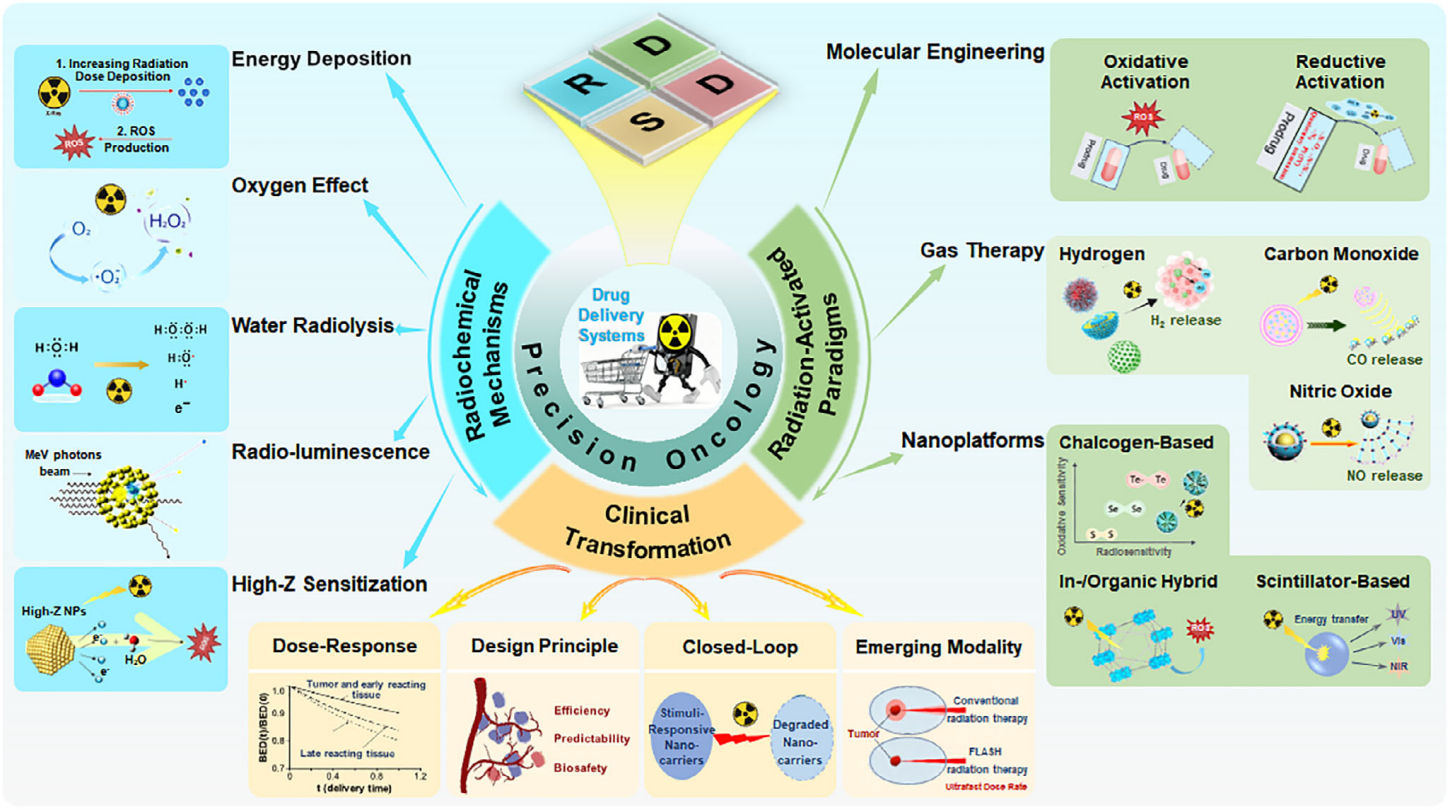
Conceptual framework and evolutionary roadmap of radiotherapy‐triggered drug delivery systems (RDDS). The framework integrates fundamental radiochemical mechanisms with radiation‐responsive molecular engineering and multifunctional nanoplatforms, providing a comprehensive design paradigm for precise drug release and gas therapy. It further outlines the translational roadmap of RDDS, highlighting dose‐correlated activation, clinically compatible design principles, and AI‐enabled closed‐loop optimization toward precision radiotherapy.

To address these challenges, we propose a unifying conceptual framework that organizes RDDS into two fundamentally distinct yet complementary paradigms: structural disassembly and molecular actuation. Structural disassembly involves radiation‐induced destabilization or degradation of carrier systems, leading to bulk release of encapsulated payloads, whereas molecular actuation operates at the covalent level, where ionizing radiation directly induces bond‐specific chemical transformations to activate prodrugs or release bioactive species without requiring carrier breakdown [29, 30]. Importantly, this distinction extends beyond mechanistic classification and directly impacts translational potential. Molecular actuation strategies—particularly those driven by electron‐mediated or direct energy transfer processes—enable activation modes that are less dependent on microenvironmental variability and more closely aligned with radiation dosimetry [31]. Building on this framework, we further introduce a translation‐oriented design principle: clinically viable RDDS must exhibit highly predictable, dose‐correlated activation behavior, in which therapeutic output scales predictably with delivered radiation dose (Figure 1). This principle provides a practical criterion for evaluating existing systems and guiding future development. In this review, we bridge radiochemistry, molecular engineering, and clinical translation, systematically covering fundamental mechanisms (Section 2), molecular actuation strategies (Section 3), nanoplatform evolution (Section 4), gas therapy extensions (Section 5), and translation‐oriented design (Section 6), ultimately positioning RDDS as programmable interfaces that convert physical radiation into controlled biochemical outcomes for precision oncology.

## 2 Radiochemical Mechanisms and Molecular Foundations

The rational design of radiation‐responsive systems requires a fundamental understanding of the interactions between ionizing radiation and biological matter [32]. Unlike photodynamic therapy, where photon absorption is mediated by specific chromophores, X‐ray interactions are inherently non‐selective and governed by the physics of energy deposition and the chemistry of water radiolysis [33].

### 2.1 Physics of Energy Deposition

**FIGURE 2:**
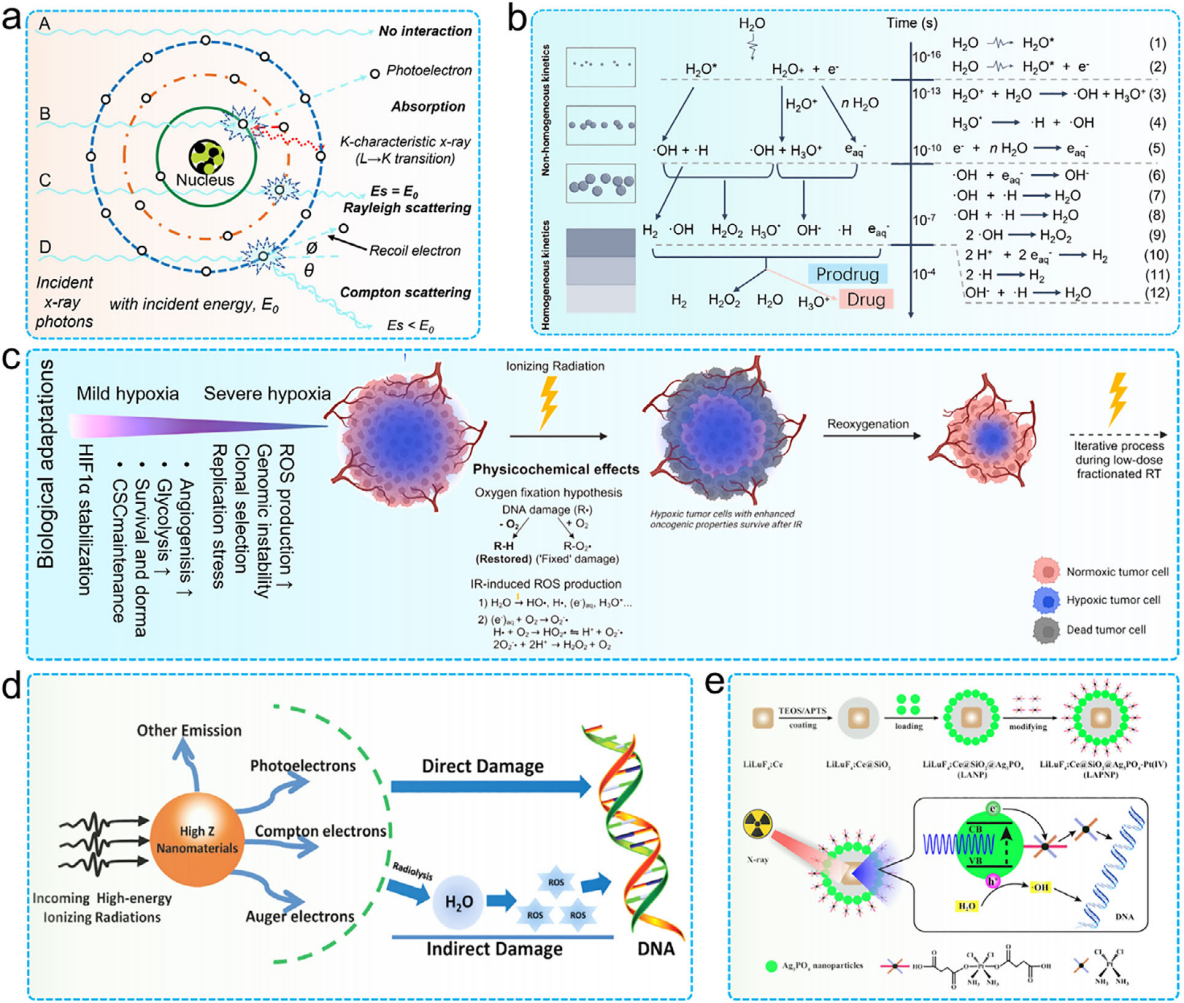
Fundamental radiochemical mechanisms underlying radiotherapy‐triggered drug delivery systems (RDDS). (a) Interaction of X‐rays and γ‐rays with matter through photoelectric absorption, Rayleigh scattering, and Compton scattering. Adapted with permission from Ref. [37]. Copyright 2005, SNMMI. (b) Time‐resolved water radiolysis illustrating the generation and evolution of reactive radiolytic species from early spur reactions to homogeneous radical diffusion. Reproduced with permission from Ref. [16]. Copyright 2025, Springer Nature. (c) Mechanisms of hypoxia‐induced radioresistance, including HIF‐1α activation, suppressed ROS generation, reduced DNA damage, and reoxygenation during fractionated radiotherapy. Adapted with permission from Ref. [43]. Copyright 2023, Elsevier Ltd. (d) Enhancement of radiation dose deposition and secondary electron production by high‐Z nanoradiosensitizers. Reproduced with permission from Ref. [36]. Copyright 2019, Wiley‐VCH. (e) X‐ray‐excited radioluminescence‐mediated photodynamic therapy using LiLuF_4_:Ce@SiO_2_@Ag_3_PO_4_@Pt(IV) nanoparticles (LAPNP). Reproduced with permission from Ref. [60]. Copyright 2018, American Chemical Society.

Upon entering biological tissue, high‐energy X‐ray photons interact with matter through three primary mechanisms, depending on photon energy [34]. At low energies (*E* < 50–100 keV), the photoelectric effect dominates, wherein photons are fully absorbed by inner‐shell electrons, resulting in photoelectron ejection and subsequent emission of characteristic X‐rays or Auger electrons. This interaction scales strongly with atomic number (∝*Z* ^3^/*E* ^3^), forming the physical basis for high‐Z radio‐sensitization [35, 36]. At intermediate energies (*E* = 100 keV–10 MeV), Compton scattering becomes predominant, generating recoil electrons and scattered photons (Figure 2a). This process is largely independent of atomic number but correlates with electron density, and thus dominates in clinical radiotherapy using megavoltage linear accelerators (6–18 MV) [34]. At higher energies (*E* > 1.02 MeV), pair production occurs, yielding electron–positron pairs through interaction with the nuclear field [37].

Collectively, these primary events produce cascades of secondary electrons (photoelectrons, Compton electrons, and Auger electrons), which are the principal agents of energy deposition in biological systems. As these electrons traverse tissue, they induce extensive ionization and excitation events [38, 39]. Given that biological tissues consist predominantly of water (∼70%–80%), most deposited energy is funneled into water radiolysis, thereby initiating the chemical processes central to RDDS.

### 2.2 Water Radiolysis and the Radical Landscape

Water radiolysis constitutes the fundamental chemical engine of RDDS, occurring across ultrafast timescales from femtoseconds to microseconds [16]. This process generates a complex mixture of reactive species, including hydrated electrons ($e_{\mathrm{aq}}^{-}$), hydroxyl radicals (·OH), hydrogen atoms (H ·), hydrogen peroxide (H_2_O_2_), and molecular hydrogen (H_2_) (Figure 2b). For low linear energy transfer (LET) radiation (e.g., X‐rays and γ‐rays), typical *G*‐values (per 100 eV) are ∼2.6–2.8 for $e_{\mathrm{aq}}^{-}$ and ·OH, ∼0.6 for H ·, and ∼0.7 for H_2_O_2_, corresponding to sub‐micromolar concentrations per Gray [40].

This radiolytic environment exhibits a distinctive redox duality. Hydroxyl radicals act as extremely potent oxidants (*E* ^0^ ≈ +2.7 V), reacting at diffusion‐controlled rates with most organic substrates via hydrogen abstraction or electrophilic addition. In contrast, hydrated electrons (*E* ^0^ ≈ −2.9 V) represent one of the strongest reducing species in aqueous systems, complemented by hydrogen atoms (*E* ^0^ ≈ −2.3 V) [41]. This coexistence of strong oxidizing and reducing species provides a versatile chemical foundation for RDDS design, enabling systems that are selectively activated through either oxidative or reductive pathways depending on the therapeutic context.

### 2.3 The Oxygen Effect and the Hypoxia Paradox

Oxygen (O_2_) is the dominant modulator of radiation chemistry in vivo [42]. Under normoxic conditions, molecular oxygen enhances radiotherapy efficacy by “fixing” radiation‐induced DNA damage and efficiently scavenging reducing species [43]. In particular, $e_{\mathrm{aq}}^{-}$ react with O_2_ at near diffusion‐limited rates (*k* ≈ 1.9 × 10^10^/m/s), forming superoxide anion radical ($O_{2}^{\cdot -}$) and thereby suppressing reductive chemistry [44]. However, solid tumors are frequently hypoxic due to abnormal vasculature and rapid proliferation [42]. This gives rise to a central paradox in RDDS design. While hypoxia reduces the effectiveness of radiotherapy—often requiring significantly higher doses—it simultaneously creates favorable conditions for reductive activation mechanisms (Figure 2c).

In hypoxic environments (typically <0.1% O_2_), the lifetime of $e_{\mathrm{aq}}^{-}$ is substantially prolonged, extending from nanoseconds to microseconds [45]. This increase expands their diffusion range from tens to several hundred nanometers, enabling the remote activation of radiation‐sensitive chemical bonds within tumor tissue [46]. Consequently, reductive pathways become inherently tumor‐selective, operating most efficiently in regions that are otherwise resistant to radiation [16, 47]. Importantly, hypoxia does not significantly affect the primary generation of ·OH but rather suppresses oxygen‐dependent radical propagation. As a result, reductive activation is enhanced, whereas certain oxidative amplification mechanisms may be attenuated—an important consideration for RDDS design [48].

### 2.4 High‐Z Sensitization: The Auger Cascade

To enhance the relatively weak interaction of X‐rays with soft tissue, high‐Z materials are widely incorporated into RDDS. Elements such as Au (*Z* = 79), I (*Z* = 53), Hf (*Z* = 72), and Bi (*Z *= 83) exhibit significantly elevated photoelectric cross‐sections, thereby enhancing energy absorption [49]. Following X‐ray absorption, inner‐shell ionization generates core vacancies, which relax via emission of cascades of low‐energy Auger electrons (Figure 2d). These electrons possess short mean free paths (<100 nm) and high LET, resulting in highly localized energy deposition [50, 51].

A critical consequence is the formation of a locally amplified radical environment, where concentrations of reactive species can exceed those in bulk solution by orders of magnitude [52, 53]. By positioning cleavable linkers or prodrugs in proximity to high‐Z nanostructures, these systems create a localized “reactive hotspot”, significantly enhancing radiation‐triggered chemical transformations at clinically relevant doses [54].

### 2.5 Radioluminescence: X‐Ray Excited Optical Luminescence

An alternative activation mechanism involves the conversion of X‐ray energy into optical photons via radioluminescence. Inorganic scintillators absorb X‐rays and re‐emit photons in the ultraviolet (UV), visible (Vis), or near‐infrared (NIR) range through X‐ray‐excited optical luminescence [55]. This process originates from the generation of electron–hole pairs within the scintillator lattice, followed by radiative recombination at dopant centers (e.g., lanthanide ions such as Tb^3+^ and Eu^3+^). The resulting emission effectively functions as an internal light source within deep tissues [56].

Radioluminescence enables the activation of conventional photo‐responsive chemistries—such as photocleavable linkers and photosensitizers—beyond the penetration limits of an external excitation source [57, 58]. For example, the UV light generated in situ can trigger *o*‐nitrobenzyl cleavage or activate photodynamic therapy pathways that would otherwise be inaccessible [59, 60]. This strategy bridges radiotherapy and photochemistry, expanding the design space of RDDS toward hybrid, multi‐modal activation mechanisms (Figure 2e).

## 3 Molecular Engineering of Radiation‐Activated Prodrugs

Radiation‐activated prodrugs (RAPs) are predicated on the rational identification of radiotherapy‐removable protecting groups that exhibit high thermodynamic stability during systemic circulation yet undergo rapid and selective transformation in the presence of radiolytic species [26]. Within the conceptual framework established in this review, the vast majority of RAP systems can be conceptualized within the paradigm of molecular actuation, wherein ionizing radiation directly induces bond‐level chemical transformations—reduction, oxidation, or elimination—to modulate drug activity with molecular precision, independent of carrier disassembly (Table 1). While this design inherently reduces reliance on stochastic multi‐step cascade reactions, offering a fundamentally more predictable and programmable therapeutic modality, activation efficiency may still be influenced by local microenvironmental factors, including endogenous reductant concentrations and oxygen availability [61].

**TABLE 1:** Radiation‐activated prodrug (RAP) systems.

Chemical motif	RAPs	Activation mechanism	Evaluation dose (Gy)	Quantitative release metric (Yield vs. Dose)	Drug	Tumor models	Background leakage	Refs.
	RABiT	Radiolytic reduction (*e* ^−^ *_aq_*)	2–10	Linear response	MMAE, DOX	TBP3743 ATC tumor	Stable	[66]
Azide	MAL‐NPs	Radiolytic reduction (*e* ^−^ *_aq_*)	2,3	R848‐N3 and MAL‐NPs were reduced to 94.51% and 90.38% at 4 Gy	R848	CT26 tumor	<20% release	[67]
	RT‐PRO	Radiolytic reduction (*e* ^−^ *_aq_*)	0, 10, 30, and 60	Linear response	PROTAC	MCF‐7 tumor	Stable	[68]
	NO‐CPT	Radiolytic reduction ((*e* ^−^ *_aq_*))	16	∼140 nm prodrug conversion per Gy	Camptothecin	HCT116 tumor	Stable	[47]
N‐oxide	NOS/NOR prodrug	Radiolytic reduction (*e* ^−^ *_aq_*))	20	99.2% for NOS and 92.6% for NOR at 40 Gy	Sorafenib; Regorafenib	Liver cancer	Stable	[71]
	0‐R848	Radiolytic reduction ((*e* ^−^ *_aq_*))	6	∼120 nm per Gy	R848	B16 tumor	Stable	[62]
	O‐IMDQ	Radiolytic reduction (*e* ^−^ *_aq_*))	6, 10	None	IMDQ	MC38 tumor	Stable	[72]
Ouaternary ammonium	QA‐CFZ	Radiolytic reduction (*e* ^−^ *_aq_*))	16	Linear response	Carfilzomib	CT26 tumor	Stable	[21]
	NAPC‐ADC	Radiolytic reduction (*e* ^−^ *_aq_*))	8,12	In vitro: 92.1% conversion at 8 Gy; in vivo: 35 nm per Gy	MMAE	4T1 tumor	Stable	[74]
Azo bond	RT‐ADC	Radiolytic reduction (*e* ^−^ *_aq_*))	2	Linear response	MMAE	SUNE2 tumor	1.30% release over 7 days	[80]
DHBC	DHBC‐MeRho_;_ DHBC‐MMAE	ROS oxidation (·OH)	0–60, 4	16 nmol per Gy for MeRho, 40 nmol per Gy for MMAE	MMAE	4T1 cells	Stable	[88]
Benzothiazoline	Amide‐containing benzothiazolines	ROS oxidation (·OH/o_2_^–^)	55, 110	∼36%/80% conversion at 55 Gy	Coumarin fluorophore	None	Stable	[91]

### 3.1 Reductive Activation: Exploiting Hydrated Electrons

Given the exceptional reducing potential of $e_{\mathrm{aq}}^{-}$ or H ·, together with their prolonged lifetime under hypoxic conditions, reductive activation represents one of the most robust and tumor‐selective RAP strategies [16, 62]. Notably, this mechanism is largely governed by radiation dose rather than the heterogeneous tumor microenvironment, rendering it particularly attractive for clinical translation [63].

#### 3.1.1 Azide‐Based Systems

Aromatic azide (─N_3_), typically embedded within phenyl or sulfonyl scaffolds, functions as a highly efficient electron acceptor [64]. Upon exposure to $e_{\mathrm{aq}}^{-}$, they are selectively reduced to aniline derivatives (─NH_2_), which subsequently undergo self‐immolative fragmentation (e.g., 1,6‐elimination) to release covalently linked payloads [65]. Owing to its high chemoselectivity and efficient radiolytic uncaging, this strategy has been extensively validated across multiple therapeutic contexts. Representative examples include albumin–drug conjugates incorporating phenyl azide linkers, which combine the prolonged circulation and tumor‐accumulation properties of serum albumin with radiation‐triggered molecular activation. The radioactivated albumin‐bound inducible therapeutic (RABiT) platform achieves more than a 130‐fold increase in active drug concentration within irradiated tumors following clinically relevant fractionated radiotherapy (2 Gy × 5 fractions), while maintaining minimal activation in non‐irradiated tissues, thereby demonstrating excellent spatial selectivity [66].

**FIGURE 3:**
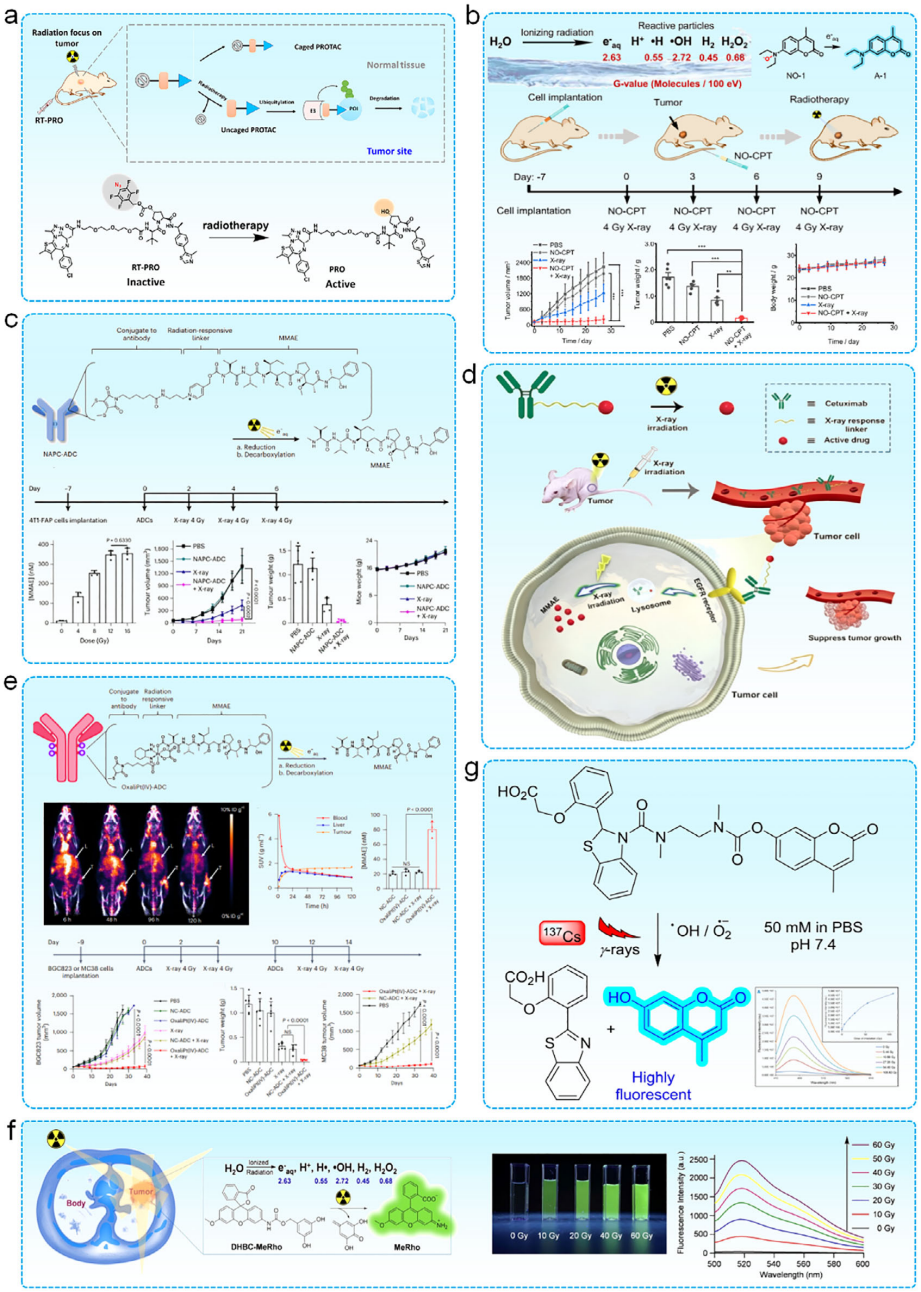
Representative molecular activation strategies for radiotherapy‐triggered drug delivery systems (RDDS). (a) Radiation‐activated RT‐PROTAC prodrug enabling X‐ray‐triggered targeted protein degradation. Reproduced with permission from Ref. [68]. Copyright 2023, American Chemical Society. (b) Hydrated electron‐mediated activation of an N‐oxide prodrug for radiotherapy‐triggered chemotherapy. Adapted with permission from Ref. [47]. Copyright 2022, American Chemical Society. (c) Radiation‐responsive N‐alkyl‐4‐picolinium caged (NAPC)‐ADC platform enabling dose‐dependent MMAE release. Adapted with permission from Ref. [74]. Copyright 2024, Springer Nature. (d) EGFR‐targeted, azobenzene‐linked antibody–drug conjugate (ADC) for X‐ray‐controlled payload release. Reproduced with permission from Ref. [80]. Copyright 2025, American Chemical Society. (e) Oxaliplatin(IV)‐based ADC platform enabling radiotherapy‐triggered dual‐drug release. Reproduced with permission from Ref. [31]. Copyright 2024, Springer Nature. (f) DHBC‐mediated radiation‐induced molecular uncaging through hydroxylation‐triggered self‐immolative cleavage. Adapted with permission from Ref. [88]. Copyright 2020, Wiley‐VCH. (g) Dose‐dependent γ‐ray activation of benzothiazoline derivatives as radiation‐responsive fluorogenic probes and prodrug scaffolds. Reproduced with permission from Ref. [91]. Copyright 2023, Wiley‐VCH.

Beyond cytotoxic agents, this strategy has been successfully extended to kinase inhibitors (e.g., pazopanib), chemotherapeutics (e.g., doxorubicin), and immune agonists (e.g., R848), all of which can be activated under clinically relevant X‐ray doses. The radiation‐activated R848 prodrug exhibited a clear dose‐dependent activation profile under clinically relevant radiation doses, producing localized immune agonist release while minimizing background activation, thereby significantly enhancing radiotherapy‐induced antitumor immunity [65, 67]. More recently, this approach has been further expanded to radiation‐activated PROTACs (RT‐PROTAC), enabling spatiotemporally controlled protein degradation at tumor sites (Figure 3a) [68, 69]. Quantitative analysis demonstrated progressive uncaging with increasing X‐ray dose (10–60 Gy). Following 30 Gy irradiation, the activated RT‐PROTAC restored antiproliferative activity (IC_50_, 122 nm), comparable to that of the parent PROTAC (IC_50_, 120.4 nm), whereas the inactive precursor exhibited substantially lower cytotoxicity (IC_50_, 1019 nm), providing representative evidence for dose‐dependent molecular activation. Importantly, because azide reduction is largely orthogonal to endogenous mammalian enzymatic pathways, these systems generally exhibit minimal background activation and excellent radiation selectivity, making aromatic azides one of the most extensively validated radiation‐responsive motifs for molecular actuation [70].

#### 3.1.2 N–Oxides‐Based Systems

N–oxide functional groups represent another well‐established class of reductively activatable motifs. Acting as efficient electron traps, N–oxides undergo reduction to regenerate the corresponding tertiary amines via electron uptake, protonation, and dehydration [71]. This transformation has been successfully applied to alkaloid‐based therapeutics, such as camptothecin derivatives, where masking the basic nitrogen enhances aqueous solubility and reduces off‐target toxicity [47]. Upon X‐ray irradiation, the active amine is efficiently restored with radiochemical yields (*G*‐values) of approximately 130–140 nm/Gy, demonstrating a dose‐correlated activation profile compatible with clinically relevant fractionated radiotherapy regimens (Figure 3b). Quantitative studies further established the dose–response relationship using N–oxide prodrugs of the multi‐kinase inhibitors sorafenib (NOS) and regorafenib (NOR). At a clinically relevant dose of 10 Gy, active drug conversion reached 56.4% for NOS and 22.3% for NOR, while increasing the radiation dose to 40 Gy resulted in near‐complete activation, yielding 99.2% sorafenib and 92.6% regorafenib release [71]. A key advantage of N–oxide‐based RAPs lies in their ability to selectively unmask pharmacologically essential nitrogen centers, thereby preserving target‐binding affinity while enabling precise spatiotemporal control of activation [47].

Furthermore, the N–oxide caging strategy has recently been extended to address the severe on‐target, off‐tumor toxicity associated with systemic immunotherapy. In the Single Atom Engineering for Radiotherapy‐Activated Prodrugs (SAE‐RAP) strategy, a single oxygen atom is introduced onto the quinoline nitrogen of Toll‐like receptor 7/8 (TLR7/8) agonists (e.g., R848), disrupting receptor‐binding interactions and reducing immunostimulatory activity by more than 4000‐fold. Upon localized X‐ray irradiation, $e_{\mathrm{aq}}^{-}$ efficiently reduce the N–oxide, restoring active R848 with an activation yield of approximately 120 nm/Gy [62]. Building upon this concept, oxygen‐masked imidazoquinoline (O‐IMDQ) prodrugs further demonstrated that localized radiotherapy‐triggered activation stimulates intratumoral dendritic cells through coordinated STING and MyD88 signaling, thereby reactivating pre‐existing CD8^+^ T cells within the tumor microenvironment. Importantly, combining these immune prodrugs with fractionated low‐dose radiotherapy (e.g., 6 Gy × 3 fractions) preserves immune cell function while maintaining efficient prodrug activation, resulting in potent local tumor control, enhanced abscopal responses, and suppression of distant metastasis [72].

#### 3.1.3 Quaternary Ammonium‐Based Systems

Quaternary ammonium (QA) structures, particularly electron‐deficient aromatic systems such as pyridinium and benzyl ammonium motifs, are capable of efficiently capturing hydrated electrons [73]. This interaction triggers single‐electron transfer processes that result in heterolytic bond cleavage and subsequent payload release. QA‐caged small‐molecule prodrugs demonstrate efficient tumor accumulation and dose‐dependent activation in vivo, such as QA‐caged carfilzomib (QA‐CFZ) prodrug. Following 16 Gy X‐ray irradiation, the released carfilzomib restores cytotoxicity to a level comparable with that of the free drug, while fractionated radiotherapy (4 Gy × 4 fractions) combined with QA‐CFZ produces significant tumor growth inhibition in murine models [21]. Beyond small molecules, N‐alkyl‐4‐picolinium (NAP)‐based linkers have been successfully incorporated into antibody–drug conjugates (ADCs) to enable radiation‐triggered release of highly potent payloads such as monomethyl auristatin E (MMAE) (Figure 3c) [74]. Quantitative studies demonstrated that a 100 nm NAP‐caged ADC achieved 92.1% ± 1.9% payload conversion following 8 Gy irradiation in vitro, whereas 12 Gy irradiation in vivo resulted in essentially complete intratumoral MMAE release, corresponding to an activation yield of approximately 35 nm/Gy. In addition to their favorable radiochemical performance, QA‐based systems offer significant synthetic advantages, as they can be readily constructed through straightforward alkylation reactions [75]. This accessibility facilitates rapid library generation and structure–activity relationship optimization.

#### 3.1.4 Azo‐Based Systems

Azo linkages (─N═N─) constitute a versatile class of reduction‐sensitive triggers that can be selectively cleaved by hydrated electrons generated during water radiolysis [76]. Mechanistically, this process proceeds via a two‐electron reduction to a hydrazine intermediate, followed by N─N bond scission driven by electron donation from adjacent substituents [77]. Importantly, the reactivity of azo bonds can be finely tuned through molecular design [78]. Incorporating electron‐withdrawing or cationic substituents and extending π‐conjugation effectively lower the LUMO energy, thereby enhancing electron affinity and accelerating reduction kinetics [79]. Recent studies have demonstrated efficient, dose‐dependent cleavage of azo‐caged compounds under clinically relevant irradiation conditions. The rationally designed AZO‐Rhodamine2 probe bearing ortho‐ and para‐hydroxyl substituents released 2.8 µm active fluorophore from an initial 5 µm prodrug following 20 Gy X‐ray irradiation, corresponding to an apparent conversion efficiency of approximately 105% relative to the theoretical hydrated‐electron yield. Moreover, intracellular activation was detectable at doses as low as 2.5 Gy and increased progressively up to 25 Gy in MOLT‐4 cells, demonstrating a clear dose‐correlated activation profile [76]. Furthermore, azobenzene‐masked camptothecin (XP‐CPT) prodrugs revealed a pronounced dose‐dependent drug release that increased with radiation dose and approached a plateau at approximately 50 Gy. At a fixed dose of 30 Gy, activation efficiency decreased with increasing prodrug concentration, indicating that radiolytically generated reactive species became rate‐limiting under high substrate loading [80]. Leveraging this controllable activation behavior, azobenzene linkers have been incorporated into epidermal growth factor receptor (EGFR)‐targeting ADCs for spatiotemporally regulated release of highly potent payloads such as MMAE. Combined with localized fractionated radiotherapy (2 Gy per fraction), these radiation‐triggered ADCs achieved >90% tumor growth inhibition (TGI) and prolonged median survival time by more than 165% in vivo compared with control groups (Figure 3d). Collectively, these features position azo‐based linkers as highly tunable platforms for radiation‐triggered drug release, with minimal interference from endogenous enzymatic processes.

#### 3.1.5 Platinum (IV)‐Based Systems

Platinum(IV) complexes represent a distinct class of inorganic RAPs that undergo reductive activation to yield cytotoxic Pt(II) species, such as cisplatin or oxaliplatin [81]. Hydrated electrons initiate this process and proceed through a two‐electron transfer mechanism involving transient Pt(III) intermediates, ultimately resulting in axial ligand dissociation and drug activation [82, 83]. Crucially, this reduction is significantly enhanced under hypoxic conditions, aligning with the tumor microenvironment and conferring intrinsic selectivity [84, 85]. Exposure of 10 µm Pt(IV) prodrug solutions to 60 Gy X‐ray irradiation resulted in approximately 80% release of the axial ligands and the corresponding Pt(II) drugs. At the cellular level, treatment with 10 µm oxaliplatin Pt(IV) prodrug followed by a clinically relevant 4 Gy irradiation generated approximately 765 nm active oxaliplatin, highlighting the compatibility of this strategy with conventional fractionated radiotherapy. This radiochemical activation translated into substantial biological efficacy, producing an approximately eightfold increase in DNA‐bound platinum after 4 Gy irradiation and inducing near‐complete tumor regression with minimal systemic toxicity in preclinical models. Furthermore, the Pt(IV) motifs have been further incorporated into targeted delivery systems, including ADCs, enabling spatially confined drug release and synergistic chemoradiotherapy (Figure 3e) [31]. These studies highlight the considerable potential of Pt(IV)‐based platforms for achieving dose‐correlated chemoradiotherapy while expanding the therapeutic applications of high‐valent metallodrugs for precision oncology [86].

### 3.2 Oxidative Activation: Harnessing Hydroxyl Radical

In contrast to reductive mechanisms, oxidative activation strategies exploit the high reactivity of hydroxyl radicals, which are generated in abundance during water radiolysis, particularly in normoxic or re‐oxygenated tumor regions. Their strong oxidizing capability enables rapid engagement with electron‐rich motifs, facilitating efficient bond cleavage and payload release [87].

#### 3.2.1 Dihydroxybenzyl Carbamate‐Based Systems

The dihydroxybenzyl carbamate (DHBC) linker is a representative ·OH‐responsive motif, designed to undergo electrophilic aromatic substitution upon radical attack, followed by self‐immolative elimination to release the caged payload [88]. This process proceeds rapidly under irradiation and has been widely adopted in radiation‐activated systems integrating chemotherapy and radiotherapy [89]. In practical implementations, DHBC linkers have been incorporated into small‐molecule prodrugs to enable localized release of chemotherapeutics such as MMAE and doxorubicin (DOX) [88], as well as integrated into metal–organic frameworks for the delivery of SN38 [90]. In a DHBC‐based radiation‐responsive fluorogenic probe, X‐ray‐induced hydroxylation triggers self‐immolative cleavage to release MeRho, yielding a linear fluorescence response over 0–60 Gy with an activation efficiency of approximately 16 nmol/Gy (Figure 3f). Furthermore, the DHBC‐MMAE RAPs achieve a higher activation efficiency of approximately 40 nmol/Gy. Under a clinically relevant 4 Gy irradiation, DHBC‐MMAE markedly restored the cytotoxicity of MMAE, reducing 4T1 cell viability to approximately 30%, whereas either the prodrug or irradiation alone produced minimal toxicity. These results demonstrated that DHBC‐mediated molecular uncaging enables predictable, dose‐dependent activation of both imaging probes and therapeutic agents under clinically relevant irradiation conditions [88]. Despite these advantages, oxidative activation via DHBC is inherently susceptible to competition from intracellular antioxidants. High glutathione (GSH) concentrations can effectively quench ·OH radicals before they engage the linker, thereby attenuating activation efficiency. This limitation highlights a central design principle: the performance of oxidative molecular switches depends not only on their intrinsic reactivity but also on their ability to compete within the cellular redox environment.

#### 3.2.2 Benzothiazolines‐Based Systems

Benzothiazoline‐based linkers constitute another class of ROS‐responsive motifs that undergo oxidative cleavage under ionizing radiation [91]. The activation mechanism is initiated by hydrogen abstraction at the benzylic position by ROS such as ·OH or $O_{2}^{\cdot -}$, leading to the formation of a stabilized benzylic radical intermediate. This intermediate is subsequently oxidized to a benzothiazolium species, which undergoes hydrolytic cleavage to release the conjugated payload, typically in the form of amines or carboxylic acids [92]. Quantitative proof‐of‐concept studies utilizing profluorescent model compounds have explicitly demonstrated a dose‐dependent release profile for this linker. Specifically, initial cleavage and subsequent release of a model coumarin fluorophore can be triggered at radiation doses as low as 5 Gy. Furthermore, the extent of activation scales quantitatively with the applied dose, achieving approximately 80% and 36% payload conversion for distinct water‐soluble benzothiazoline derivatives under a 55 Gy irradiation (Figure 3g) [91]. Moreover, the concomitant formation of fluorescent benzothiazole derivatives provides an intrinsic optical readout of activation, enabling real‐time monitoring of drug release processes and offering potential for theranostic integration. From a design perspective, benzothiazoline linkers exhibit favorable responsiveness to ROS while maintaining structural stability under physiological conditions [93]. However, similar to other oxidative systems, their activation efficiency may be influenced by local oxygen availability and antioxidant concentrations, reinforcing the importance of microenvironment‐aware molecular engineering.

## 4 Radiation‐Responsive “All‐in‐One” Nanoplatforms

Compared with small‐molecule RAPs, which often undergo rapid renal clearance and exhibit limited tumor retention, nanomedicine‐based systems provide prolonged circulation, EPR‐mediated accumulation, and the capacity for multicomponent integration. These features enable the construction of “all‐in‐one” platforms that co‐deliver radiosensitizers, therapeutic agents, and imaging functionalities within a single architecture (Table 2) [94]. From a conceptual standpoint, two mechanistic paradigms govern radiation‐responsive nanomedicine. The first is structural disassembly, in which irradiation induces physicochemical destabilization of the carrier, resulting in bulk release of encapsulated payloads [95]. The second is molecular actuation, where radiation triggers well‐defined chemical transformations at specific functional sites [96]. While historically treated as distinct strategies, an important convergence is now emerging: advanced nanoplatforms increasingly integrate molecularly defined reaction centers within structurally dynamic carriers, thereby achieving both pharmacokinetic control and chemical precision.

**TABLE 2:** Radiation‐responsive “all‐in‐one” nanoplatforms.

DDS format	Nanomaterials	X‐ray dosage (Gy)	Response mechanism	Tumor models	Refs.
Chalcogen‐based nanocarriers	Se‐NPs	2	X‐ray‐triggered reactive species: ─Se─Se‐cleavage	4T1 tumor	[29]
	Se‐polymer	2	X‐ray and ROS triggered reactive species: ─Se─Se‐cleavage	4T1 tumor	[99]
	PNG/DOX	5	X‐ray‐triggered reactive species: ─Se─Se‐cleavage	A549 tumor	[100]
	PEG‐Se NPs	8	X‐ray‐triggered reactive species: ─Se─Se‐cleavage	HeLa cells	[101]
	GE11‐THZ1‐NPS	2	X‐ray and ROS‐triggered reactive species: ─Se─Se‐ cleavage	U‐CH1 Tumor	[102]
	PSeR/DOX	5, 2.5	X‐ray‐triggered reactive species ─Se─Se‐cleavage	MDA‐MB‐231 tumor	[104]
	CH@M0N@D0X	1	X‐ray‐triggered reactive species ─Se─Se‐cleavage	4T1 tumor	[105]
	DMSNs@A0	6	X‐ray‐triggered reactive species: ─Se─Se‐cleavage	Cervical cancer	[106]
	BTe‐Pd‐Au‐R	4	X‐ray and 980 nm laser triggered reactive species: Group cleavage	CT26 tumor	[108]
	Ru‐GNC	2	X‐ray‐triggered photoelectron energy transfer	4T1 tumor	[114]
Inorganic–organic hybrid nanosystems	Au‐LAHP‐vDOX	8	X‐ray triggered cascade	U87 MG tumor	[115]
	Au nanoparticles	1, 2, 3, 5, and 10	X‐ray and UV Irradiations triggered high‐Z atoms to produce ROS	N/A	[116]
	VP + Au@Liposomes	4	X‐ray triggered high‐Z atoms to produce ROS	Colorectal cancer	[117]
	DOX‐Hb‐Lipo	8	X‐ray triggered high‐Z atoms to produce ROS	Melanoma tumor	[119]
	Au‐R	2	X‐ray‐activated secondary electron	4T1 tumor	1121]
	Hf‐DBP‐R	2	X‐ray‐triggered high‐Z atoms to produce ROS	CT26 and MC38 tumor	[124]
	Hf‐TP‐SN	2	X‐ray‐triggered reactive species: Group cleavage	CT26 and 4T1 tumor	[89]
	Hf‐DBP‐QP‐SN	2	X‐ray‐triggered reactive species: Group cleavage	CT26 and 4T1 tumor	[90]
	PWCu nanocapsules	6	X‐ray‐triggered cuproptosis	4T1‐R tumor	1128]
	SciDD	8	X‐ray activated UV scintillating	TBP3743 and CT26 colon tumor	[134]
	ZnS‐A NPs	6	X‐ray activated UV scintillating	U87 MG tumor	[135]
	RBS‐T‐SCNPs	6	X‐ray activated UV scintillating	A549 tumor	[136]

### 4.1 Chalcogen‐Based Nanocarriers

**FIGURE 4:**
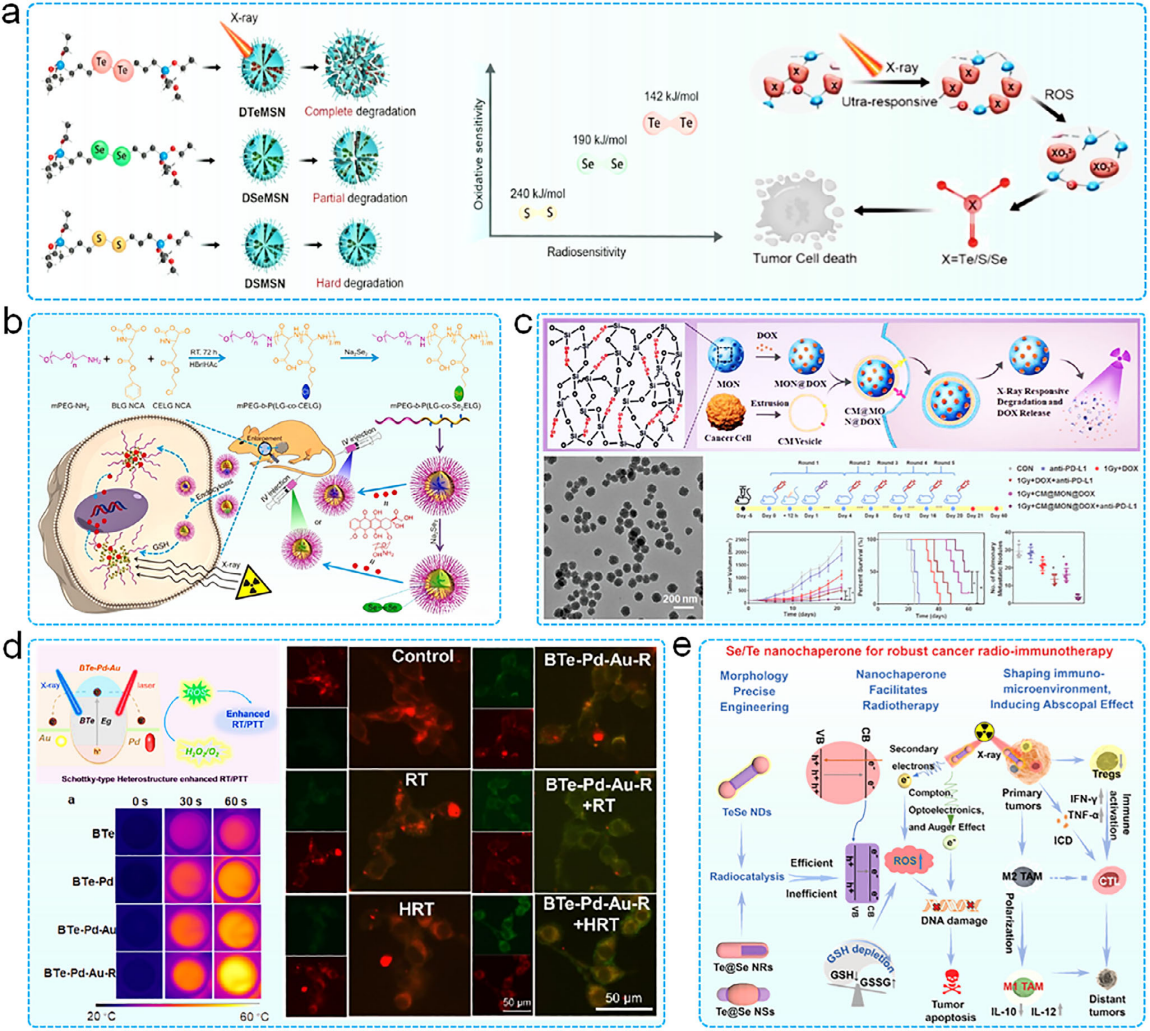
Representative chalcogen bond‐engineered nanoplatforms for precision radiotherapy‐triggered drug delivery systems (RDDS). (a) Chalcogen bond‐engineered nanoplatform with bond‐dependent X‐ray responsiveness (Te─Te > Se─Se > S─S). Adapted with permission from Ref. [97]. Copyright 2024, Wiley‐VCH. (b) Diselenide‐crosslinked polypeptide nanogel (PNG) enabling ROS‐mediated Se─Se bond cleavage and X‐ray‐triggered doxorubicin release. Reproduced with permission from Ref. [100]. Copyright 2021, Elsevier Ltd. (c) Diselenide‐bridged mesoporous organosilica nanoparticles (MONs) for low‐dose X‐ray‐triggered drug release and synergistic chemo‐immunotherapy. Adapted with permission from Ref. [105]. Copyright 2020, Wiley‐VCH. (d) BTe─Pd─Au chalcogen‐based nanosystem enabling X‐ray/NIR‐responsive ROS generation for synergistic radio‐photothermal therapy. Adapted with permission from Ref. [108]. Copyright 2021, American Chemical Society. (e) TeSe nanoheterojunctions as morphology‐engineered radiosensitizers for enhanced ROS generation, tumor microenvironment remodeling, and radio‐immunotherapy. Reproduced with permission from Ref. [98]. Copyright 2023, Wiley‐VCH.

Chalcogen elements (S, Se, and Te) provide a chemically versatile platform due to their redox‐active covalent bonds (Figure 4a), whose dissociation energies decrease systematically down the group (S─S ≈ 240 kJ/mol > Se─Se ≈ 172 kJ/mol > Te─Te ≈ 126 kJ/mol) [97]. This trend directly correlates with increasing susceptibility to radiolysis‐derived ROS, enabling tunable responsiveness across different radiation dose regimes [98].

#### 4.1.1 Diselenide‐Containing Systems

Diselenide bonds are particularly attractive due to their intermediate bond strength, which balances circulatory stability with radiation sensitivity. Under X‐ray irradiation, ROS generated via water radiolysis oxidize Se─Se linkages into hydrophilic seleninic acid (R─SeO_2_H) or selenone species, driving a hydrophobic‐to‐hydrophilic transition that destabilizes nanostructures such as polymeric micelles and triggers drug release at relatively low doses (<5 Gy) [29, 99, 100, 101]. DOX‐loaded polypeptide nanogels (PNGs) exhibited minimal drug leakage over 48 h without irradiation, but released approximately 36% of their payload after a 5 Gy X‐ray dose, reaching 53% release within 8 h when the dose was escalated to 50 Gy (Figure 4b) [100]. It is important to note that the classification of chalcogen‐mediated activation within our analytical framework depends on the structural context in which the bond is employed. When diselenide bonds serve as cleavable linkers in small‐molecule prodrugs, ROS‐mediated oxidation directly releases the active payload through a covalent bond transformation and therefore represents a typical example of molecular actuation. In contrast, when diselenide bonds are incorporated into the backbone or crosslinking network of polymeric nanocarriers, bond cleavage induces large‐scale physicochemical changes, including carrier destabilization and cargo release. In this scenario, the primary mechanism of drug release arises from carrier breakdown and is therefore classified as structural disassembly. This distinction illustrates how the same radiochemical event can produce fundamentally different therapeutic outcomes depending on the structural level at which it is engineered.

Furthermore, beyond physical carrier breakdown, these diselenide‐based nanocarriers often represent a unique dual‐mode paradigm. On one level, drug release is governed by structural disassembly, as carrier destabilization leads to bulk cargo liberation. On another level, the oxidation products themselves exhibit intrinsic bioactivity, introducing a layer of molecular actuation independent of the delivered drug [102]. For instance, quantitative studies on selenium‐containing nanoparticles confirmed that a 5 Gy radiation dose induced approximately 50% DOX release at 24 h. Beyond drug release, the resulting seleninic acid actively downregulated HLA‐E, acting synergistically with the radiotherapy (Combination Index < 1) to enhance natural killer cell‐mediated cytotoxicity. Notably, these nanoparticles also exhibited high tumor targeting, delivering nearly 4% of the administered dose to solid tumor tissues [103, 104].

Recent advances have leveraged this dual functionality to construct increasingly sophisticated systems. Diselenide‐bridged mesoporous organosilica nanoparticles (MONs) enable co‐delivery of chemotherapeutics and immune checkpoint inhibitors (Figure 4c), thereby coupling cytotoxicity with immune activation [105, 106]. This design demonstrated a burst‐like release of 45.2% of loaded DOX within the first 6 h under an ultra‐low dose of 1 Gy (in the presence of 100 µm H_2_O_2_), compared to only 18.1% background release without radiation. Under 1 Gy of X‐ray irradiation, the IC_50_ of the membrane‐coated MONs was 2.1‐fold lower than that of the uncoated counterparts. When combined with PD‐L1 immune checkpoint blockade, half of the treated mice became completely tumor‐free and survived up to 60 days post‐tumor challenge, demonstrating profound inhibition of both primary tumor growth and pulmonary metastasis with minimal off‐target cardiovascular or hepatic toxicity [105]. Core‐crosslinked architectures further improve systemic stability by shielding Se─Se bonds within hydrophobic domains, while preserving rapid degradation upon localized irradiation. Beyond chemotherapy, emerging designs exploit selenium biology itself—for example, selectively amplifying ROS production in tumor cells while promoting selenoprotein‐mediated protection in normal tissues, thereby mitigating radiation‐induced toxicity [107]. Despite these promising attributes, challenges remain in precisely controlling selenium dosage and understanding its interaction with endogenous selenium metabolism pathways. Addressing these issues will be essential for optimizing therapeutic windows and advancing clinical translation.

#### 4.1.2 Tellurium‐Containing Systems

Tellurium‐based nanocarriers extend chalcogen chemistry toward the extreme of radiation responsiveness. The low Te─Te bond energy (∼126 kJ/mol) renders these systems highly susceptible to oxidative cleavage, while the relatively high atomic number of tellurium confers intrinsic radio‐sensitization through enhanced X‐ray absorption (Figure 4d) [108]. Quantitative comparisons reveal that optimizing tellurium into specific heterostructures significantly boosts its therapeutic index. For instance, engineering Te into palladium and gold heterostructures (BTe‐Pd‐Au‐R) improves the sensitization enhancement ratio (SER) up to 2.10 under a clinical 4 Gy X‐ray dose, substantially outperforming bare Te nanorods (SER = 1.11) and reducing cancer cell viability to 27.6%.

Two complementary mechanisms dominate Te‐based systems. First, ROS‐driven oxidation converts Te (II) species into hydrophilic Te═O derivatives, triggering carrier disassembly and drug release. This mechanism is often exploited in polymeric or organosilica frameworks, where Te‐containing linkages serve as both structural elements and responsive triggers. In addition to X‐ray responsiveness, tellurium platforms exhibit exceptional photothermal capabilities [109]. For example, double‐camouflaged Te nanoparticles (RS─Te) achieved a high photothermal conversion efficiency of 33.8%. Under 808 nm laser irradiation (1.5 W/cm), these systems rapidly raised localized tumor temperatures to 59.3°C, plummeting cancer cell viability to a mere 8.03% (Figure 4d). Second, heterojunction‐mediated charge transfer—particularly in Te/Se hybrid nanostructures—enhances electron–hole separation under X‐ray irradiation, thereby amplifying ·OH generation via improved radiocatalytic efficiency [98]. Structural engineering plays a critical role in determining this conversion yield. The dumbbell‐shaped TeSe nanoheterojunctions (TeSe NDs) demonstrate 1.8‐fold, threefold, and sixfold higher radiosensitization efficacies compared to core–shell Te@Se nanorods, bare Te nanorods, and Se nanoparticles, respectively, achieving up to 60% late cell apoptosis at a standard 4 Gy X‐ray dose (Figure 4e).

Notably, Te‐containing nano‐prodrugs can couple degradation with therapeutic output. For example, Te─Te cleavage may simultaneously release cytotoxic tellurite species (TeO_3_^2−^) and co‐loaded immunomodulatory ions such as Mn^2+^, which activate the STING pathway [110]. This integration of chemical degradation and immune activation exemplifies the multifunctionality achievable in chalcogen‐based systems. However, the very reactivity that underpins their responsiveness also introduces translational challenges, particularly in maintaining stability during circulation while ensuring efficient activation at the tumor site [111].

### 4.2 Inorganic–Organic Hybrid Nanosystems

**FIGURE 5:**
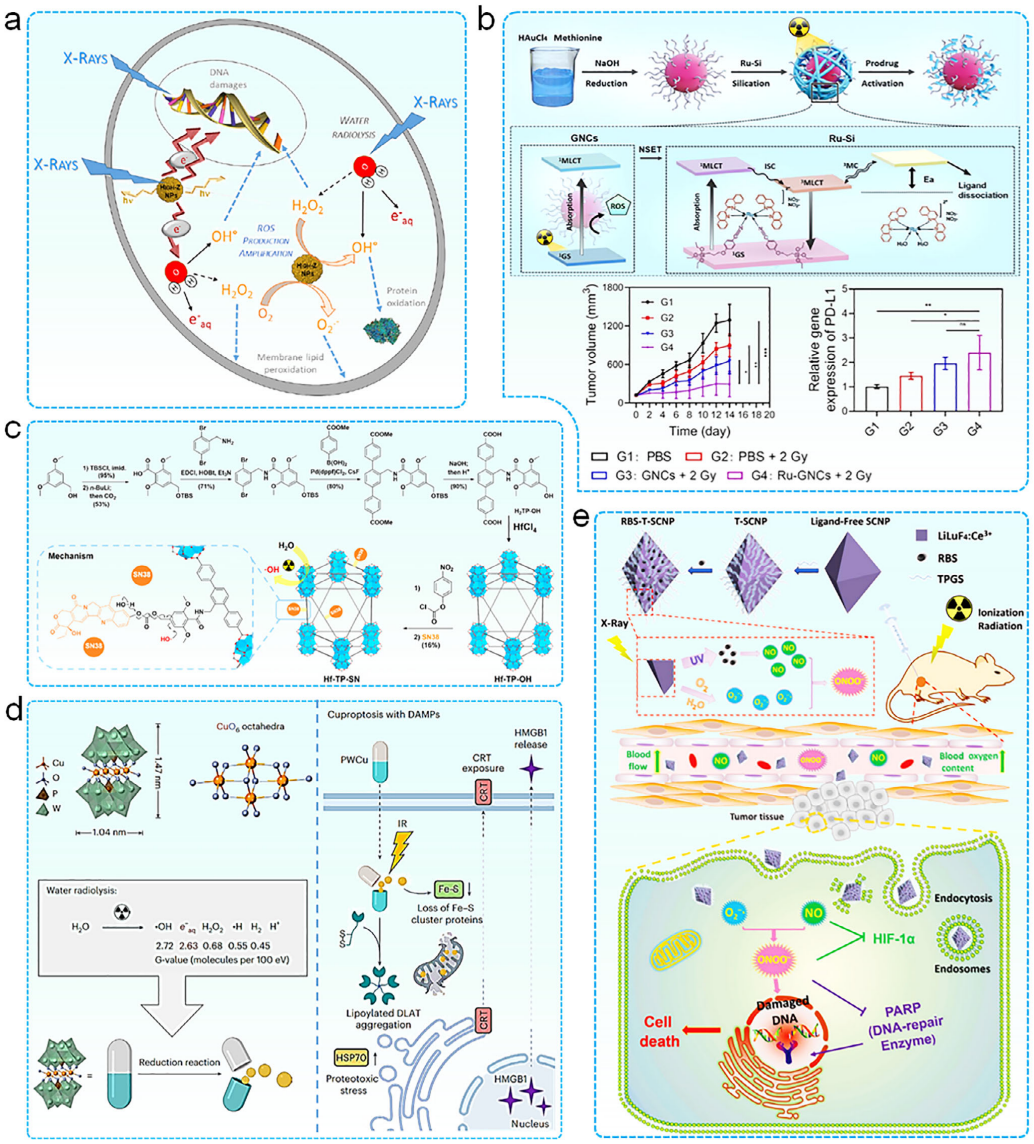
Representative high‐Z element‐based hybrid nanosystems for radiotherapy‐triggered drug delivery systems (RDDS). (a) High‐Z metal‐based nanoparticles enhancing radiotherapy through secondary electron generation, ROS amplification, and DNA damage. Reproduced with permission from Ref. [50]. Copyright 2018, Elsevier B.V. (b) Ru‐GNC nanoplatform enabling X‐ray‐triggered prodrug activation and PD‐L1‐mediated immunomodulation. Adapted with permission from Ref. [114]. Copyright 2023, Wiley‐VCH. (c) Hf‐based nanoscale metal–organic framework (Hf‐TP‐SN nMOF) enabling X‐ray‐triggered release of SN38 through postsynthetic prodrug modification. Reproduced with permission from Ref. [89]. Copyright 2023, American Chemical Society. (d) PWCu nanocapsules enabling hydrated electron‐mediated Cu^+^ release and irradiation‐induced cuproptosis. Adapted with permission from Ref. [128]. Copyright 2024, Springer Nature. (e) RBS‐T‐SCNP nanoplatform for X‐ray‐triggered peroxynitrite (ONOO^−^) generation and on‐demand radiotherapy. Reproduced with permission from Ref. [136]. Copyright 2018, Wiley‐VCH.

Hybrid nanosystems combine high‐Z inorganic cores with functional organic components, leveraging enhanced photoelectric absorption to amplify local energy deposition (Figure 5a) [50]. This leads to increased generation of hydrated electrons and ROS, thereby lowering the radiation dose required for therapeutic activation [112, 113].

#### 4.2.1 Gold Nano‐Hybrids

Gold‐based nanosystems provide a particularly instructive platform for dissecting the mechanistic landscape of X‐ray‐triggered drug activation [23]. Broadly, two fundamentally distinct pathways can be delineated: (i) ROS‐dependent structural disassembly, in which radiation‐induced reactive species indirectly trigger carrier degradation, and (ii) nano‐surface energy transfer (NSET)‐driven molecular actuation, where energy is directly transferred from the irradiated gold core to adjacent prodrug moieties [114]. These two mechanisms are not merely operationally different, but represent distinct paradigms in how radiation energy is transduced into therapeutic activity—one mediated by stochastic chemical intermediates, the other governed by highly predictable, dose‐correlated photophysical processes [115].

In ROS‐mediated systems, gold nanoparticles act as potent radiosensitizers that amplify local radiolysis, generating high fluxes of hydroxyl radicals (·OH) and related ROS [116]. These radicals subsequently oxidize carrier components—such as unsaturated lipid membranes or polymer backbones—resulting in membrane disruption, structural collapse, and burst release of encapsulated payloads [117, 118, 119]. While this mechanism benefits from radical amplification and can achieve robust therapeutic outputs, it is intrinsically constrained by the tumor microenvironment. High intracellular concentrations of GSH and other antioxidants compete directly for ·OH, while local oxygen tension modulates radical propagation and lifetime [120]. As a consequence, drug release efficiency becomes strongly context‐dependent, introducing variability that decouples the relationship between administered radiation dose and actual pharmacological output—an important limitation for clinical standardization and reproducibility.

By contrast, NSET‐driven systems establish a direct and quantitatively predictable coupling between radiation energy deposition and drug activation. Upon X‐ray irradiation, gold nanoclusters generate cascades of high‐energy photoelectrons that transfer energy to nearby prodrug moieties (e.g., Ru or Pt complexes) via a distance‐dependent mechanism (∝1/*r* ^4^) [114]. Recent evaluations of a core‐shell nanoplatform (Ru‐GNC) demonstrate an exceptional NSET efficiency of ∼92.5%. Operating independently of ROS, the system ensures negligible background leakage in H_2_O_2_/GSH‐rich environments but achieves >70% active Ru(II) release under a 4 Gy X‐ray dose. This precise activation significantly enhances therapeutic potency, yielding an in vitro IC_50_ of 23.34 µg Au/mL against 4T1 cells—3.9 times more potent than irradiated bare gold. Furthermore, in vivo treatment with a low 2 Gy dose amplified the intratumoral CD8^+^/CD4^+^ T‐cell ratio 7.3‐fold. When paired with a PD‐L1 blockade, the therapy effectively halted metastasis by decreasing immunosuppressive regulatory T‐cells (Tregs) in distant tumors 1.7‐fold (Figure 5b) [114]. While this mechanism substantially reduces the reliance on diffusible ROS intermediates compared to conventional oxidative pathways, it is important to note that its activation efficiency is not completely immune to microenvironmental variables. The successful coupling of physical dose to chemical release remains strictly modulated by the requirement for ultra‐short donor‐acceptor distances (typically <10 nm), the precise spatial localization of the prodrug on the nanoparticle surface, and potential interference from competitive microenvironmental scavengers that may intercept direct electron transfer [121]. Despite these constraints, by mitigating the impact of macroscopic variations in hypoxia or antioxidant enzyme activity, NSET‐based platforms exhibit a dosimetry‐governed activation profile, in which therapeutic output scales directly with radiation input.

#### 4.2.2 Hf/Zr Nano‐MOFs

Nano–metal–organic frameworks (nMOFs) constitute a versatile class of X‐ray‐responsive platforms that integrate high‐Z inorganic clusters (e.g., Hf_6_ or Zr_6_ nodes) with photoactive organic linkers such as porphyrins or BODIPY derivatives, thereby enabling coupled control over radiation energy deposition and downstream chemical reactivity within a single architecture [122, 123]. Upon X‐ray irradiation, the high‐Z clusters act as nanoscale antennas that enhance local dose deposition via the photoelectric effect, promoting water radiolysis and the generation of ROS, particularly ·OH. Concurrently, energy can be transferred to adjacent photosensitizing ligands to initiate radiotherapy–radiodynamic therapy (RT–RDT), resulting in singlet oxygen (^1^O_2_) production [124]. Notably, the mechanism of this energy coupling remains unresolved: activation may proceed either through direct excitation of the linker by secondary/Auger electrons or via indirect ROS‐mediated pathways. The lack of clear mechanistic discrimination between these routes represents a critical limitation, as it constrains the rational optimization of energy transfer efficiency and ROS quantum yield in nMOF design.

Functionally, nMOFs enable multiple, partially orthogonal drug release pathways that integrate molecular actuation with structural disassembly [125]. Covalently conjugated therapeutics linked via ROS‐labile groups (e.g., 3,5‐dimethoxybenzyl carbonate) undergo oxidative cleavage followed by 1,4‐elimination, enabling precise molecular‐level activation of payloads such as SN38 or gasotransmitters (e.g., CO, H_2_S) [126]. In parallel, physically encapsulated molecules can be released through ROS‐induced framework perturbation or pore diffusion, introducing a carrier‐level release component [127]. This dual‐mode behavior places nMOFs at the interface between covalent bond actuation and macroscopic carrier disruption. Importantly, these mechanistic features translate into robust therapeutic performance in vivo: representative systems such as Hf‐TP‐SN (Figure 5c) and Hf‐DBP‐QP‐SN achieve pronounced TGI at clinically relevant low radiation doses (2–6 Gy), with complete regression observed in some models and minimal systemic toxicity [89, 90]. Specifically, the Hf‐TP‐SN nanoplatform, with an SN38 conjugation rate of ∼16%, released 1.35% of its payload after 10 Gy X‐ray irradiation, representing a fivefold improvement over the free prodrug. This activation produced dose‐modifying ratios at 10% survival (DMR_10%_) values of 2.566 (CT26) and 2.663 (MC38), while fractionated radiotherapy (3 × 2 Gy) achieved TGI of 96.5% in CT26 and 88.9% in 4T1 models, with complete tumor eradication in 40% of CT26‐bearing mice. Similarly, the mixed‐ligand Hf‐DBP‐QP‐SN platform (∼25% SN38 conjugation) achieved a 2.4% SN38 conversion yield in the presence of 0.5 mm H_2_O_2_ under X‐ray irradiation, corresponding to an 18.7‐fold enhancement over the free prodrug. This translated into a Radiation Enhancement Factor (REF_10_) of 2.52, a 13.9‐fold increase in ROS generation, and TGI values of 93.5% (CT26) and 95.2% (4T1) under the same (3 × 2 Gy) regimen.

Beyond chemotherapy delivery, the intrinsic modularity of nMOFs further enables seamless integration of additional therapeutic modalities. For instance, the SHF@PMOF platform couples ROS generation with gas therapy upon irradiation to induce mitochondrial dysfunction—characterized by ATP depletion, Ca^2+^ dysregulation, and NADH inhibition—thereby amplifying radio‐sensitivity [22]. A 6 Gy X‐ray dose significantly enhances the release of CO and H_2_S by 6.70‐ and 5.46‐fold, respectively, compared to the gas donor alone. This localized dual‐gas burst severely disrupts cellular metabolism, evidenced by a sharp drop in intracellular ATP (from 6.87 to 2.67 nm) and a 3.28‐fold reduction in NADH levels. This targeted metabolic interference directly amplifies radiosensitivity, reducing cancer cell survival to just 31%—a 2.58‐fold increase in cytotoxicity compared to non‐irradiated controls—thereby achieving highly efficient tumor suppression at low radiation doses. Collectively, these features position nMOFs as a uniquely adaptable platform that leverages both the physical precision of radiotherapy and the chemical tunability of framework design to achieve spatiotemporally controlled, multi‐modal therapeutic responses.

#### 4.2.3 W‐Cu Nanocapsules

PWCu nanocapsules represent a distinct class of radiation‐responsive systems in which therapeutic activity arises directly from radiation‐induced transformation of the material itself, rather than from the release of an encapsulated payload. Structurally, these nanocapsules consist of polyoxoanion frameworks exposing highly accessible Cu^2+^ centers, enabling direct capture of radiolytically generated $e_{\mathrm{aq}}^{-}$ [128]. Upon X‐ray irradiation, $e_{\mathrm{aq}}^{-}$ produced from water radiolysis reduced Cu^2+^ to Cu^+^ in a dose‐dependent manner [129]. The resulting Cu^+^ species function as the active cytotoxic mediator, inducing cuproptosis through mitochondrial metabolic disruption, including aggregation of lipoylated proteins in the tricarboxylic acid (TCA) cycle and destabilization of Fe─S cluster‐containing enzymes. This mechanism is distinct from apoptosis or ferroptosis, exploiting unique metabolic liabilities in cancer cells [130]. Unlike conventional carrier‐based systems, the copper center simultaneously serves as both the radiation‐responsive substrate and the therapeutic effector. Mechanistically, this activation pathway closely parallels the radiolytic reduction of Pt(IV) prodrugs to cytotoxic Pt(II) species. In both systems, radiolytically generated reducing species are captured by a transition‐metal center, producing a biologically active lower‐valence state. The primary difference lies in the downstream pharmacological response rather than the activation mechanism itself. Whereas Pt(IV) complexes generally require axial ligand dissociation to release an active chemotherapeutic agent, the radiolytically generated Cu^+^ species directly mediates cuproptosis. By integrating the high‐Z radiation‐harvesting element W and the responsive Cu center within a single nanostructure, PWCu nanocapsules facilitate localized electron transfer and provide a representative example of reductive metal‐center activation.

From a therapeutic perspective, PWCu nanocapsules exhibit pronounced efficacy against radioresistant tumors. Following fractionated radiotherapy, residual tumor cells upregulate cuproptosis‐related regulators such as FDX1 and LIAS, rendering them hypersensitive to copper‐induced cytotoxicity [131]. By exploiting this acquired metabolic vulnerability, PWCu systems selectively eliminate resistant tumor populations. Concurrently, treatment induces immunogenic cell death, as evidenced by the release of DAMPs (e.g., CRT, HMGB1, HSP70), which promotes CD8^+^ T cell infiltration and systemic antitumor immunity (Figure 5d) [128]. Under X‐ray irradiation, PWCu nanocapsules efficiently generated cytotoxic Cu^+^ species at a clinically relevant dose of 4 Gy, exhibiting an IC_50_ of 1.02 µm and a REF of 1.88. This activation also induced a robust systemic antitumor immune response, increasing CD4^+^ and IFNγ^+^CD8^+^ T‐cell infiltration in distant tumors by 4.14‐ and 6.25‐fold, respectively. Consequently, PWCu suppressed tumor recurrence and metastasis, and extended median survival from 56 to 90 days with a 40% cure rate in a re‐irradiation model (2 Gy × 3). Collectively, these features position PWCu nanocapsules as a paradigm of molecular actuation‐driven radiotherapy, transforming radiation from a purely physical insult into a biologically targeted and mechanistically precise therapeutic modality.

#### 4.2.4 Scintillator‐Based Nanocarriers

Scintillator‐based nanocarriers introduce an orthogonal activation paradigm by converting deeply penetrating X‐ray radiation into localized UV or visible photons, thereby enabling classical photochemical reactions in tissues beyond the reach of external light sources [132, 133, 134]. In these systems, scintillating nanoparticles (e.g., ZnS, LaF_3_:Ce, LiLuF_4_:Ce) function as energy transducers that bridge ionizing radiation and molecular photochemistry, effectively coupling radiotherapy with light‐driven chemical transformations at the nanoscale [60]. Upon irradiation, the emitted photons activate adjacent photosensitive components through multiple mechanistic pathways. These include: (i) photoisomerization processes such as *trans*‐to‐*cis* conversion of azobenzene moieties, which disrupt *π–π* stacking and increase membrane permeability to facilitate drug diffusion [135]; (ii) photocleavage of caged linkers (e.g., nitroveratryl oxycarbonyl groups), enabling controlled release of cytotoxic payloads such as MMAE [134]; and (iii) activation of gas‐releasing compounds (e.g., Roussin's black salt), generating NO and downstream reactive species such as peroxynitrite (ONOO^−^) [136]. Notably, these mechanisms support dose‐dependent and temporally programmable release profiles, allowing synchronization with clinically relevant fractionated radiotherapy regimens.

These mechanistic advantages translate into robust in vivo therapeutic performance. Representative systems such as PETAzo@ZnS‐A/DOX achieve ∼90% drug release and 94.31% tumor growth inhibition in glioblastoma models, significantly outperforming free drug controls [135]. The SciDD platform enables efficient MMAE activation at low radiation doses (≈2 Gy), yielding >600‐fold increases in intratumoral active drug concentration and broad efficacy across tumor models [134]. Importantly, diffusible reactive intermediates generated during activation produce pronounced bystander effects, partially mitigating the impact of heterogeneous nanoparticle distribution. Additional platforms (e.g., RBS‐T‐SCNPs) further enhance radio‐sensitization by inducing DNA damage, suppressing repair pathways, and alleviating hypoxia, achieving sensitizer enhancement ratios up to 1.81 (Figure 5e) [136]. Collectively, scintillator‐based systems integrate the spatial precision of radiotherapy with the chemical versatility of photochemistry, offering a compelling strategy for spatiotemporally controlled, multi‐modal cancer therapy with reduced systemic toxicity.

## 5 Radiation‐Driven Gas Therapy

A rapidly emerging frontier in RDDS is the in situ generation of therapeutic gases (gasotransmitters) under ionizing radiation. These small, diffusible molecules—including nitric oxide, carbon monoxide, and hydrogen—exert pleiotropic effects on tumor biology, such as modulation of vascular tone, disruption of mitochondrial function, and enhancement of radio‐sensitivity (Table 3) [137, 138, 139]. Unlike conventional chemotherapeutics, gasotransmitters are not delivered as stable payloads but are instead generated on demand, offering an opportunity to achieve precise spatiotemporal control over bioactive signaling events. In this context, X‐ray irradiation serves as a uniquely advantageous trigger, providing deep tissue penetration and clinically established dosimetric control [138]. Importantly, the design of radiation‐driven gas therapy systems can be systematically interpreted through the framework of molecular actuation versus carrier‐mediated release, a distinction that critically influences activation fidelity and therapeutic predictability.

**TABLE 3:** Radiation‐driven gas therapeutic systems.

Nanomaterials	Forms of gas	X‐ray dosage (Gy)	Response mechanism	Tumor models	Refs.
Bi‐SNO NPs	NO	5	X‐rays break down the S─N of S‐nitrosothiol through the high‐Z effect	U14 tumor	[138]
PEG‐USMSs‐SNO	NO	1, 3, 5, and 10	X‐rays directly trigger S─N cleavage	4T1 tumor	[144]
ZGOMn‐RFJS	NO	0.0009	X‐rayexcited persistent luminescence activates the NO donor via an energy transfer	4T1 tumor	[145]
GW/MnCO@PLGA	CO	6	X‐ray‐triggered ROS through high‐Z effect to oxidize the Mn center and break the Mn─CO	A549 tumor	[137]
FeCO‐NRs	CO	2	X‐rays/H_2_O_2_ jointly break the Fe─CO	MC38 colon cancer	[150]
MnCO‐Tw‐SCNP	CO	6	X‐ray excited UV scintillating photochemically cleaves the Mn─CO	HeLa tumor	[153]
GM‐R848	CO	2	X‐rays break the Fe─CO and disintegrate the nanostructure	CT26 tumor	[155]
Au‐Ti0_2_@ZnS	H_2_	1	X‐rays triggered electron–hole pairs and spatial charge separation direct electrons to active surface sites, thereby reducing protons in water to H_2_	MC38 tumor	[139]

### 5.1 Nitric Oxide (NO)

**FIGURE 6:**
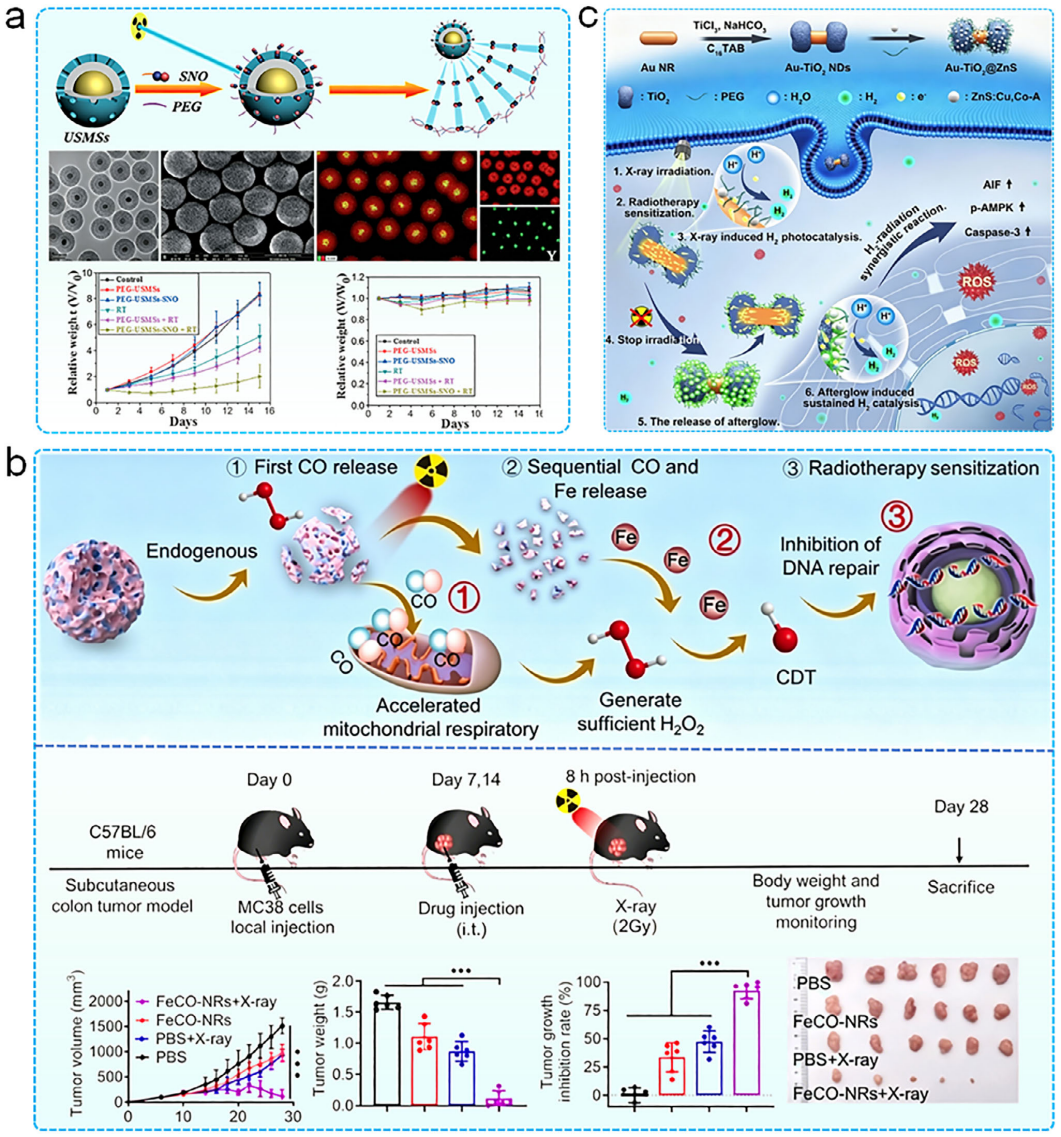
Radiation‐triggered gas‐therapeutic nanoplatforms for precision radiotherapy‐triggered drug delivery systems (RDDS). (a) PEG‐USMSs‐SNO nanoplatform enabling X‐ray‐triggered nitric oxide (NO) release for enhanced radiotherapy. Adapted with permission from Ref. [144]. Copyright 2015, Wiley‐VCH. (b) FeCO‐NRs enabling sequential X‐ray‐responsive carbon monoxide (CO) and Fe^2+^ release for synergistic chemodynamic‐radiotherapy. Adapted with permission from Ref. [150]. Copyright 2023, Elsevier Ltd. (c) Au–TiO_2_@ZnS nanoplatform promoting X‐ray‐driven electron transfer and localized hydrogen generation for radiosensitization. Reproduced with permission from Ref. [139]. Copyright 2021, Wiley‐VCH.

Nitric oxide exhibits a well‐established concentration‐dependent duality in tumor biology [140]. At high concentrations (>1 µm), NO exerts potent antitumor activity by inducing apoptosis and generating oxidative and nitrosative stress [141]. Concurrently, it functions as an effective radiosensitizer by mimicking molecular oxygen to “fix” radiation‐induced DNA damage and by improving tumor perfusion through vasodilation [142]. Realizing these therapeutic benefits, however, requires the ability to achieve localized, high‐concentration NO release with precise temporal control [143]. X‐ray radiation provides an ideal external trigger for this purpose [144]. Two principal activation strategies have been developed: (i) direct radiolytic cleavage of S‐nitrosothiols (R–SNO), exploiting the relatively low S─N bond dissociation energy (∼150 kJ/mol), and (ii) indirect activation via X‐ray‐excited scintillating nanomaterials, which convert ionizing radiation into UV/visible photons to trigger photosensitive NO donors [145]. Among these, the direct S─N bond scission pathway represents a canonical example of molecular actuation (Figure 6a) [144]. S‐nitrosothiol‐grafted mesoporous silica nanoparticles exhibited dose‐dependent NO release, producing approximately 11–12 µm NO within 1 h following 10–20 Gy X‐ray irradiation, while showing negligible background release. Notably, this ROS‐mediated activation remained effective under hypoxic conditions, enabling NO‐mediated radiosensitization. In vivo, the combination of these nanocarriers with radiotherapy effectively suppressed 4T1 tumor growth without detectable systemic toxicity.

Here, the radiation stimulus acts directly on a defined covalent bond within a molecular prodrug, liberating NO with a yield that is, in principle, quantitatively correlated with the delivered radiation dose. This mechanistic distinction carries important implications. In contrast to carrier‐dependent strategies—whose performance is modulated by tumor oxygenation and intracellular antioxidant capacity (e.g., GSH)—direct bond actuation transforms NO release into a dosimetry‐governed pharmacokinetic process [146]. In other words, NO generation can be prescribed and modulated through radiotherapy dose fractionation, reducing dependence on heterogeneous tumor microenvironmental factors [147]. This conceptual framework extends broadly across gas therapy modalities, as discussed below. Once released, NO engages in synergistic, multi‐pathway antitumor activity. Beyond directly inducing apoptosis, it participates in a powerful chemical amplification cascade: NO reacts rapidly with $O_{2}^{\cdot -}$, abundantly generated during radiotherapy, to form ONOO^−^. This highly reactive species induces extensive secondary damage to DNA, proteins, and mitochondrial components, thereby amplifying cytotoxicity and generating pronounced bystander effects [148]. Notably, the efficiency of this cascade is critically dependent on the precise spatial and temporal colocalization of NO with radiation‐induced radicals—an outcome inherently enabled by molecular actuation‐based release mechanisms.

### 5.2 Carbon Monoxide (CO)

Carbon monoxide has recently gained attention as a therapeutic gas in oncology, primarily due to its ability to disrupt tumor metabolism and sensitize cancer cells to radiotherapy [149]. Mechanistically, CO suppresses glycolysis by downregulating key metabolic enzymes, including GLUT1, HK2, and LDHA, thereby partially reversing the Warburg effect [150, 151]. Advanced nanoplatforms have been developed to precisely regulate radiation‐triggered CO release. For example, FeCO‐bridged mesoporous silica nanoreservoirs (FeCO‐NRs) exhibit sustained, X‐ray‐ and H_2_O_2_‐responsive CO release with negligible background leakage. Under clinically relevant irradiation (2 Gy), these nanoreservoirs continuously release CO and Fe^2+^, resulting in enhanced mitochondrial dysfunction and immunogenic cell death (Figure 6b) [150]. CO induces mitochondrial dysfunction, characterized by loss of membrane potential, reduced ATP production, and Ca^2+^ dyshomeostasis, ultimately promoting apoptosis and immunogenic cell death (ICD) [152]. This synergistic metabolic exhaustion drastically improves the therapeutic index. Quantitatively, FeCO‐NRs reduced the IC_50_ against MC38 cells to 60.3 µg/mL under 2 Gy irradiation, representing a 3.5‐fold improvement over conventional FeCO nanoparticles and yielding a Combination Index of 0.65. In vivo, combining FeCO‐NRs with fractionated radiotherapy (2 Gy per fraction) achieved near‐complete tumor suppression, with 66% of treated mice exhibiting complete tumor regression.

To achieve controlled CO delivery, X‐ray‐responsive nanosystems have been engineered based on two dominant strategies. The first relies on high‐Z radiosensitizers (e.g., Au nanoclusters or Hf‐based frameworks) to amplify ROS generation under irradiation, which subsequently triggers CO release from adjacent metal carbonyl complexes [137]. The second employs scintillating nanoparticles to convert X‐rays into UV/visible light, thereby activating photo‐responsive CO donors such as Mn_2_(CO)_10_ [153]. While both strategies enable spatially confined gas release, they differ fundamentally in their dependence on intermediate reactive species versus photophysical activation pathways [23, 154]. Functionally, CO‐based systems operate through a synergistic radio–gas therapeutic mechanism. Radiotherapy induces primary DNA damage, while CO simultaneously compromises cellular bioenergetics and mitochondrial integrity, impairing the ability of tumor cells to repair radiation‐induced lesions [155]. This dual assault amplifies oxidative stress and promotes apoptosis. In advanced designs, additional layers of functionality are incorporated—for example, coupling CO release with Fenton‐like reactions mediated by co‐released metal ions, or integrating co‐delivery of other gasotransmitters such as H_2_S [156]. Collectively, these strategies enable multi‐modal, spatiotemporally controlled therapeutic interventions that are particularly effective against glycolysis‐driven radio‐resistance.

### 5.3 Hydrogen (H_2_)

Hydrogen represents a mechanistically distinct gasotherapeutic agent, characterized by its selective antioxidant, anti‐inflammatory, and pro‐apoptotic effects [157]. Unlike NO and CO, which primarily act through cytotoxic and metabolic disruption pathways, H_2_ modulates intracellular signaling networks, including AMPK, apoptosis‐inducing factor, and caspase‐3, thereby influencing cell fate decisions in a more regulatory manner [158, 159]. To enable targeted H_2_ generation, X‐ray‐responsive catalytic platforms have been developed. A representative example is the Au─TiO_2_@ZnS heterostructure, in which the Au─TiO_2_ junction catalyzes hydrogen evolution under irradiation, while the surrounding long‐afterglow ZnS component enables sustained post‐irradiation release [139]. This design effectively decouples the timing of H_2_ production from continuous irradiation, allowing prolonged therapeutic action within deep tissue environments (Figure 6c). Under a low X‐ray dose of 1 Gy, the Au─TiO_2_@ZnS system generated H_2_ at a rate of 120.2 µmol/g/h, while exhibiting negligible background H_2_ production in the absence of irradiation. Owing to the persistent luminescence of ZnS, H_2_ generation was sustained for nearly 10 min after a 5‐min X‐ray exposure.

The therapeutic efficacy of H_2_ arises from its synergistic interplay with radiotherapy. On one hand, the high‐Z Au component enhances radiation‐induced ROS production and DNA damage; on the other, H_2_ selectively scavenges excessive pro‐survival radicals, mitigates inflammatory responses, and activates multiple apoptotic pathways [160]. This dual functionality enables a balanced modulation of oxidative stress—enhancing tumor cell killing while reducing collateral damage to normal tissues. In vivo, such systems have demonstrated tumor suppression rates exceeding 90%, highlighting their potential to overcome radio‐resistance while minimizing side effects [161].

## 6 Translation‐Oriented Design: From Proof‐of‐Concept to Clinical Systems

**FIGURE 7:**
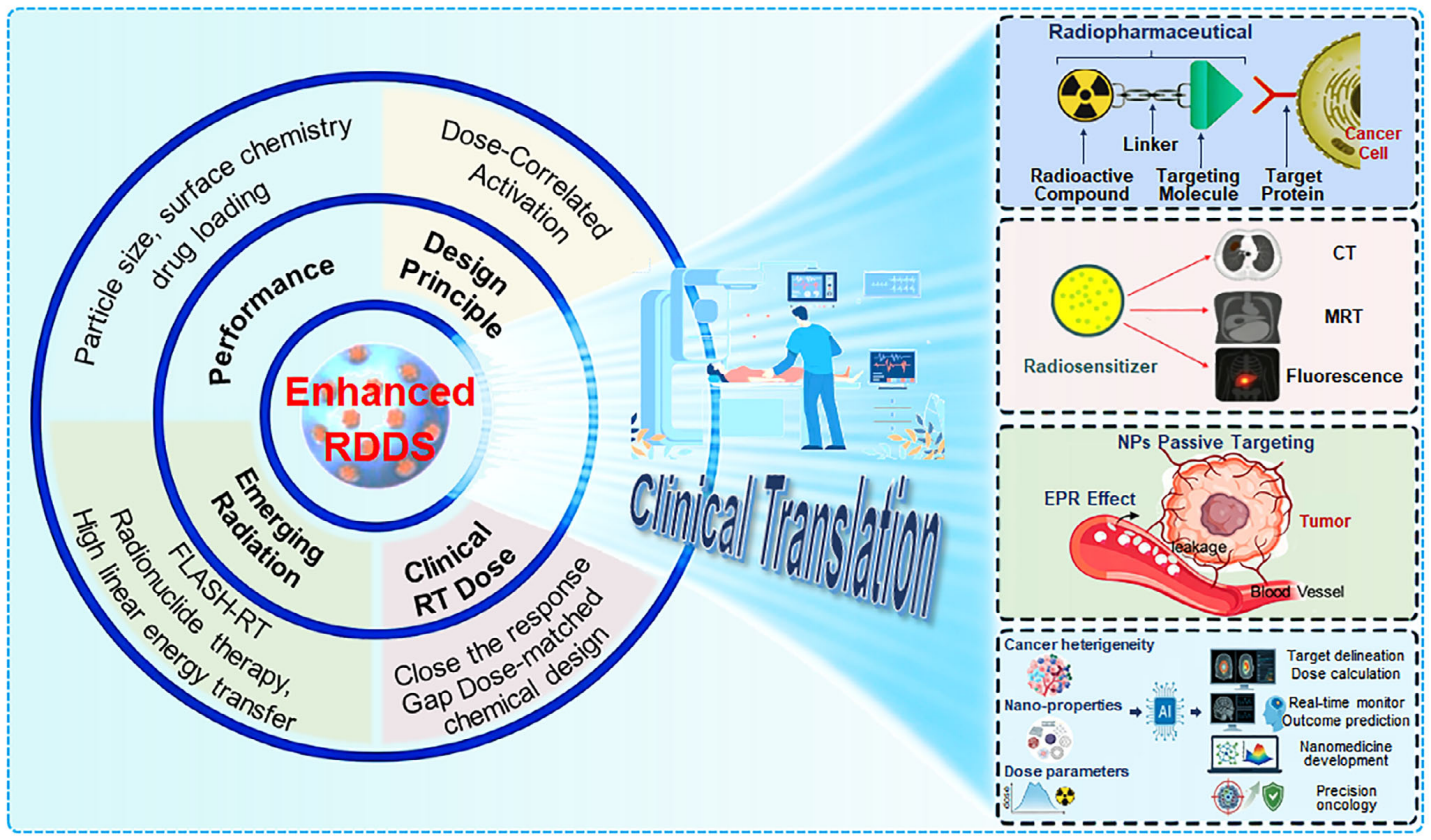
Future perspectives and translational roadmap for precision radiotherapy‐triggered drug delivery systems (RDDS). The framework outlines the key requirements for clinical translation, including dose‐correlated activation, radiotherapy dose‐matched molecular design, and optimized systemic performance. It further highlights emerging strategies for next‐generation RDDS, encompassing tumor microenvironment modulation, multimodal imaging‐guided therapy, precision targeting, and AI‐enabled closed‐loop optimization toward personalized radiotherapy.

Despite rapid advances in RDDS, the majority of reported platforms remain confined to proof‐of‐concept demonstrations, with limited pathways toward clinical implementation. This disconnect arises not from a lack of responsiveness, but from a fundamental misalignment between material design and the operational realities of radiotherapy [162]. In clinical settings, radiation dose, fractionation, and spatial delivery are tightly constrained and cannot be adapted to accommodate system‐specific activation requirements [163]. Consequently, translational RDDS must be engineered to function within predefined therapeutic windows while maintaining predictable, dose‐correlated behavior. In this section, we outline a translation‐oriented framework that addresses three central challenges: the dose–response gap under clinical irradiation, the necessity of dosimetry‐governed activation mechanisms, and the integration of RDDS into clinically and technologically viable treatment systems (Figure 7).

### 6.1 Clinical Constraints and the Dose–Response Gap

A fundamental principle governing the clinical translation of RDDS is that radiotherapy is a highly standardized modality with limited flexibility in dose delivery [164]. Contemporary clinical protocols, including fractionated regimens of 1.8–2.2 Gy per fraction and advanced delivery techniques such as IMRT and SBRT, are optimized through decades of clinical validation to balance tumor control with normal tissue toxicity [165]. These constraints define a non‐negotiable operational window within which any radiation‐responsive system must function. Within this context, the RDDS design is inherently restricted by the requirement to operate under predefined clinical dose conditions. In this sense, radiotherapy defines the operational boundary conditions to which all RDDS designs must conform.

This requirement imposes a critical limitation: the dose–response gap between the radiation dose required to activate chemical or nanostructured systems and the dose that can be safely administered to patients. At clinically relevant doses, the absolute yield of radiolytic species generated through water radiolysis is intrinsically low, typically in the sub‐micromolar range per Gray [26]. While sufficient to induce DNA damage, this radical flux is often inadequate to drive efficient cleavage of conventional chemical linkages or to trigger large‐scale structural disassembly of nanocarriers [24]. As a result, many RDDS platforms fail to achieve meaningful activation within clinically acceptable irradiation conditions [166, 167].

This mismatch explains a recurring pattern observed across early RDDS studies: robust activation under high‐dose irradiation (≥5–10 Gy), but limited efficacy under clinically relevant fractionation. For instance, disulfide‐ and early diselenide‐based systems frequently require elevated radiation doses to achieve appreciable degradation, while ROS‐dependent lipid or polymer carriers often exhibit incomplete drug release due to competition from intracellular antioxidants such as GSH [168, 169]. These findings underscore a fundamental disconnect between laboratory‐scale responsiveness and clinical feasibility, highlighting that activation efficiency observed under idealized conditions does not necessarily translate into therapeutic applicability.

Addressing this bottleneck requires a shift toward dose‐matched chemical and physical design. Chemically, the incorporation of low bond dissociation energy motifs—such as tellurium‐based linkages—enables efficient activation even under modest radical flux, allowing structural transitions at doses as low as ∼2 Gy [170]. Physically, the integration of high‐Z elements (e.g., Au, Hf) enhances local energy deposition through photoelectric interactions and Auger cascades, effectively amplifying radical generation at the nanoscale without increasing the macroscopic dose [171]. In parallel, emerging electron‐driven mechanisms—including hydrated electron‐mediated reduction and nano‐surface energy transfer—provide an alternative pathway that bypasses stochastic radical diffusion and directly couples radiation energy deposition to molecular transformation [172, 173].

To bridge the dose–response gap, RDDS designs must be specifically tailored to the intended clinical radiotherapy regimen, as the dose delivered per fraction defines the chemical activation threshold [174]. Conventional fractionation (1.8–2.2 Gy per fraction) presents the greatest challenge because of the relatively low radical flux generated during each treatment fraction, necessitating highly sensitive activation mechanisms such as low bond‐dissociation‐energy chalcogen linkages or highly efficient catalytic nanoclusters capable of maximizing radiolytic yields [166]. In contrast, hypofractionated regimens (2.5–5 Gy per fraction) provide a favorable operating window for intermediate‐responsive systems, including diselenide‐containing materials, N‐oxide prodrugs, and transition‐metal‐based NSET platforms, which can exhibit robust activation within this dose range [29, 47, 114]. SBRT (8–20 Gy per fraction) delivers ablative doses in a limited number of fractions, generates substantial bursts of ROS and secondary electrons, thereby creating an ideal environment for RDDS platforms that require higher activation thresholds, such as the ROS‐responsive polymer or lipid nanoplatforms that may remain insufficiently activated under conventional fractionation [117, 118].

Collectively, these strategies establish a clear translational criterion: RDDS platforms must be intrinsically compatible with clinical dose constraints. Systems requiring supra‐clinical irradiation are unlikely to achieve clinical adoption, making closure of the dose–response gap a defining requirement for the field.

### 6.2 Dose‐Correlated Activation as a Design Principle

Beyond achieving activation within clinically relevant dose ranges, a more fundamental requirement for RDDS is predictability. In clinical radiotherapy, absorbed dose is a precisely quantified and spatially controlled parameter. For RDDS to be compatible with this framework, drug activation must exhibit a proportional and reproducible relationship with delivered radiation dose [175]. This requirement introduces a critical distinction between stochastic, microenvironment‐dependent activation and highly predictable, dose‐governed activation. From a strict radiation physics perspective, it must be acknowledged that all primary energy deposition events—such as photoelectric absorption and Compton scattering—are inherently stochastic processes governed by Poisson statistics. However, at the macroscopic pharmacological level, a critical distinction emerges between systems whose activation depends heavily on secondary microenvironmental variables (stochastic ROS pathways modulated by oxygen and antioxidants) [176] and those coupled directly to primary radiophysical events (e.g., NSET or direct electron capture) [26]. The latter, while stochastically triggered at the atomic level, yields a macroscopic chemical output that is highly predictable and linearly correlated with the prescribed radiation dose, which we term dose‐correlated activation. The hallmark of such systems is their ability to demonstrate a robust, linear dose‐response relationship where quantitative metrics—such as drug release per Gy or activation yield per Gy—remain consistent and predictable across different fraction sizes.

In most early‐generation systems, activation is mediated by ROS generated through water radiolysis [177]. While highly reactive, the effective utilization of these species is inherently stochastic. The probability that a given radical will react with a target bond depends on competing processes, including diffusion, recombination, and scavenging by intracellular antioxidants such as GSH. In addition, oxygen concentration modulates radical propagation, introducing further variability across tumor regions [178]. At the clinical level, this mechanistic stochasticity manifests as a lack of reproducible mapping between prescribed radiation dose and pharmacological output, leading to substantial heterogeneity in drug release even under identical irradiation conditions. This stochasticity represents a fundamental limitation for clinical translation, as tumors receiving the same prescribed dose may experience markedly different levels of drug activation, thereby undermining reproducibility and complicating dose optimization [179].

In contrast, a distinct class of activation mechanisms is emerging in which radiation energy deposition is directly coupled to molecular transformation. These include hydrated electron‐mediated reduction, nano‐surface energy transfer, and radioluminescence‐driven photochemistry [180]. In such systems, activation proceeds through defined energy transfer or electron capture processes, reducing reliance on diffusible radical intermediates. Consequently, the extent of drug activation becomes more closely correlated with absorbed dose, enabling a highly predictable activation profile. This mechanistic divergence provides a basis for stratifying RDDS strategies: ROS‐dependent systems, while responsive in controlled environments, remain highly sensitive to microenvironmental fluctuations; hypoxia‐enhanced reductive systems offer partial improvement but retain dependence on oxygen heterogeneity; in contrast, platforms based on direct electron capture, NSET, or radioluminescence‐mediated activation are hypothesized to offer the potential for a more robust coupling between physical dose and chemical output [181]. It is crucial to recognize, however, that the distinction between stochastic ROS‐mediated activation and direct molecular actuation is not a strict binary, and the assertion of a robust coupling remains a highly promising hypothesis rather than a universally established conclusion across platforms. Translating this theoretical framework into consistent quantitative reality requires meeting stringent boundary conditions. For instance, NSET and radioluminescence demand precise spectral overlap and ultra‐short donor–acceptor distances, while direct electron capture is highly sensitive to local scavenger concentrations and substrate accessibility in the complex tumor microenvironment.

Nevertheless, by reducing the reliance on diffusible reactive intermediates, these direct mechanisms theoretically shift the activation profile further along the spectrum of predictability, offering the potential for a dose‐correlated relationship between physical energy deposition and chemical output. This shift supports the formulation of a clinically relevant design principle: RDDS systems must prioritize dose‐correlated activation to be compatible with modern radiotherapy [167]. Importantly, this principle reframes the central objective of RDDS design—from maximizing responsiveness to achieving quantitative predictability, whereby drug activation can be prescribed and modulated through radiation planning [26]. In this context, radiotherapy evolves from a purely cytotoxic modality into a dosimetry‐governed actuator of molecular pharmacology, and RDDS function as transducers that convert physical energy deposition into controlled biochemical outcomes.

### 6.3 From System Integration to Closed‐Loop RDDS

While molecular‐level design principles define the intrinsic capabilities of RDDS, their clinical translation ultimately depends on successful integration into the broader radiotherapy ecosystem, encompassing treatment workflows, regulatory frameworks, and emerging data‐driven technologies. From a clinical perspective, RDDS must operate compatibly across diverse radiotherapy modalities, each characterized by distinct spatial and temporal dose delivery patterns. In IMRT, highly conformal dose distributions enable spatially selective activation within tumors, whereas SBRT and hypo‐fractionated regimens impose constraints on activation kinetics due to elevated dose per fraction delivered over limited sessions. Proton and carbon ion therapies further introduce depth‐controlled energy deposition and high LET, favoring activation mechanisms requiring localized high‐energy density. In contrast, internal radionuclide therapy delivers continuous low‐dose‐rate irradiation, necessitating RDDS designs responsive to cumulative dose rather than discrete radiation fractions.

Beyond spatial compatibility across radiotherapy modalities, temporal coordination emerges as an equally critical constraint [182]. Across these modalities, a central requirement is the synchronization of radiation delivery with drug activation kinetics. RDDS should be engineered to release therapeutics within the temporal window of radiation‐induced biological vulnerability, thereby maximizing therapeutic synergy [183]. Failure to achieve such synchronization may substantially reduce therapeutic efficacy even when both radiotherapy and drug components are individually effective. Temporal coordination therefore represents a design parameter comparable in importance to chemical sensitivity and spatial targeting, reinforcing that RDDS function not as isolated materials but as time‐resolved components within a dynamic treatment system.

Crucially, the successful clinical translation of systemically administered RDDS depends on minimizing background activation while maintaining efficient responsiveness to irradiation. Although high sensitivity to radiolytic species is desirable, reductively activated chemistries, such as Pt(IV) complexes and certain N‐oxide derivatives, remain susceptible to endogenous biological reductants and hypoxia‐associated enzymatic pathways [81]. Such radiation‐independent activation may lead to premature drug release and off‐target toxicity, thereby compromising the spatiotemporal precision of radiotherapy‐triggered drug delivery. Moreover, improved local tumor control may not necessarily translate into prolonged progression‐free survival (PFS) or overall survival (OS) if background toxicity limits treatment tolerability or prevents the concurrent use of systemic therapies. Therefore, achieving true radiation selectivity requires minimizing biological activation while maintaining efficient radiation responsiveness. Future studies should incorporate more rigorous controls, including quantitative evaluation of 0 Gy background activation under physiologically relevant conditions and non‐irradiated tumor‐bearing models, to clearly distinguish biological leakage from genuine radiation‐triggered drug release [184].

Beyond achieving radiation selectivity, successful clinical translation also requires careful consideration of administration strategy, manufacturing feasibility, and clinically meaningful therapeutic benefit. Systemically administered RDDS must satisfy stringent Chemistry, Manufacturing, and Controls (CMC) requirements, including formulation stability, reproducible manufacturing, predictable biodistribution, and well‐defined renal and/or hepatic clearance pathways to minimize long‐term systemic accumulation [185]. In contrast, locally administered systems (e.g., intratumoral injection) can mitigate many challenges associated with systemic delivery but are generally limited to accessible lesions and may inadequately address infiltrative tumor margins or disseminated disease. From a clinical oncology perspective, contemporary radiotherapy already provides excellent local control for many solid tumors, making it inherently challenging to demonstrate meaningful incremental benefit in clinical trials. Furthermore, improved local tumor control does not necessarily translate into prolonged PFS or OS when distant metastasis, rather than local recurrence, represents the principal mode of treatment failure [186]. Consequently, future RDDS development should increasingly emphasize integration with systemic therapeutic strategies, such as immune checkpoint blockade and other combination therapies, to convert localized radiochemical activation into durable systemic antitumor responses.

In addition to these pharmacological and clinical considerations, successful translation further depends on regulatory approval and scalable manufacturing. RDDS are typically classified as combination products, integrating drug, device, and sometimes biological components, with regulatory pathways determined by the primary mode of action. Systems driven by chemical activation are evaluated under pharmacological frameworks, whereas those functioning as radiosensitizers or physical enhancers may be regulated as devices, introducing complexity in approval and validation. Simultaneously, manufacturing scalability imposes stringent requirements on reproducibility and quality control [187]. Because RDDS performance depends sensitively on nanoscale features such as particle size, surface chemistry, and drug loading, even minor variations can significantly alter pharmacokinetics and activation efficiency. Achieving batch‐to‐batch consistency under Good Manufacturing Practice (GMP) conditions therefore necessitates robust fabrication strategies, including microfluidic synthesis and modular self‐assembly.

Once these foundational constraints are satisfied, the next frontier lies in system‐level optimization through data‐driven feedback and artificial intelligence (AI), enabling a transition from static to adaptive treatment paradigms [188]. In conventional implementations, RDDS operate in an open‐loop mode, where radiation delivery and drug activation are decoupled and preprogrammed. By contrast, a closed‐loop framework incorporates real‐time feedback to dynamically link these processes. For this closed‐loop paradigm to operate in physical reality, AI and machine learning algorithms must be anchored to quantifiable material properties that serve as actionable data inputs, rather than relying solely on static anatomical imaging. To achieve this, RDDS must be designed to emit dynamic physicochemical signals upon irradiation. For example, radioluminescent optical dosimetry can provide real‐time, voxel‐level quantification of energy deposition, as the in situ photon emission from scintillating nanocarriers scales directly with the localized X‐ray dose [189]. Similarly, MRI chemical shift variations post‐cleavage offer a direct, non‐invasive readout of molecular actuation, enabling algorithms to map the spatial distribution of activated prodrugs versus intact precursors in deep tissue [190]. Furthermore, dynamic changes in photoacoustic signals (reflecting localized oxygen depletion or ROS generation) or PET radiotracer retention kinetics (altered upon the structural disassembly of nanocarriers) can feed predictive models with continuous temporal data [191]. By ingesting these specific, material‐derived feedback signals, AI frameworks can predictively adjust subsequent radiation parameters—such as fraction sizes, beam angles, and dose rates—thereby truly transitioning radiotherapy from a static physical treatment into an adaptive, precision‐guided pharmacological intervention [192]. Ultimately, the clinical success of AI‐assisted RDDS platforms will depend not merely on efficient radiation responsiveness, but on the integration of quantifiable and biologically meaningful feedback signals that faithfully report activation status, drug release, and therapeutic response. Anchoring intelligent algorithms to these dynamic physicochemical metrics could enable coordinated optimization across radiation physics, chemical activation, and adaptive therapeutic feedback, thereby establishing a truly integrated closed‐loop therapeutic system.

Looking forward, optimizing the clinical utility of RDDS will require integrating radiochemical design with advanced medical physics. Rather than functioning as standalone agents, future RDDS will need to be coupled with existing clinical treatment planning systems and AI‐driven pharmacokinetic modeling [193]. By utilizing real‐time spatial dosimetry to dynamically map in vivo drug release, clinicians could theoretically adjust radiation fractionation schedules on‐the‐fly to match the specific depletion and accumulation kinetics of the nanocarriers. Establishing this type of data‐driven, closed‐loop feedback—grounded in current quantitative imaging and dosimetric technologies—represents a feasible and critical next step toward precision radiochemotherapy.

### 6.4 Adapting RDDS to Emerging Radiation Modalities

While conventional low‐LET X‐rays serve as the standard benchmark for RDDS activation, the clinical landscape is rapidly evolving to include diverse and advanced radiation modalities that fundamentally alter the underlying radiochemistry. The efficiency and design requirements of RDDS are profoundly impacted when exposed to these alternative sources.

FLASH radiotherapy delivers radiation at ultra‐high dose rates (>40 Gy/s), triggering massive and instantaneous radiolytic events [194]. A defining characteristic of FLASH is the rapid depletion of local oxygen (transient hypoxia), which dramatically shifts the spatiotemporal distribution of reactive species. By instantaneously consuming oxygen, FLASH limits the propagation of oxidative species (e.g., ROS) while significantly prolonging the lifetime and diffusion distance of reducing species, such as hydrated electrons [195]. Consequently, RDDS platforms relying on oxidative pathways (e.g., ROS‐cleavable polymers) may exhibit altered activation kinetics under FLASH conditions. In contrast, molecular actuation platforms based on reductive activation mechanisms, such as N–oxide or quaternary ammonium prodrugs, may be particularly well suited to exploit this transient reductive environment.

Unlike the relatively sparse ionization events characteristic of X‐rays and γ‐rays, heavy charged particles deposit energy densely along a defined track, culminating in the Bragg peak. This high LET produces highly localized radical clusters with substantially increased radical densities compared with low‐LET photons. Such conditions can significantly alter radical recombination behavior and local reaction kinetics [196]. As a result, RDDS activation within the Bragg peak may be enhanced, potentially enabling the triggering of chemical transformations that are less efficiently activated under conventional photon irradiation, including within hypoxic tumor regions.

In contrast to the discrete fractions used in external‐beam radiotherapy, radionuclide therapy (using β‐ or α‐emitting isotopes) delivers continuous low‐dose‐rate irradiation over extended periods. Under these conditions, RDDS should be designed to respond to cumulative radiation exposure rather than transient radical bursts [197]. Moreover, because radionuclide emissions have limited path lengths, efficient activation requires precise spatial colocalization between the radionuclide source and the responsive therapeutic system, such as through direct isotope incorporation into the nanocarrier. Recent advances in Radionuclide‐induced Drug Engagement for Release (RAIDER) provide a promising strategy for adapting RDDS to internal radiation sources by using targeted radiopharmaceuticals (e.g., ^99m^Tc or ^177^Lu) to activate caged prodrugs directly at disease sites [198]. Unlike external‐beam radiotherapy, this approach exploits the systemic distribution and tumor‐targeting capability of radiopharmaceuticals to enable radiation‐triggered drug activation in disseminated tumors, thereby improving therapeutic selectivity while extending RDDS beyond localized treatment.

## 7 Conclusion

The development of RDDS reflects a broader transition in oncology from passive drug administration toward externally controllable, precision pharmacology. In this review, we have established a unified conceptual framework that organizes RDDS into two fundamental paradigms—structural disassembly and molecular actuation—and connects their radiochemical origins with molecular engineering strategies and system‐level design. Across diverse material platforms, a consistent insight emerges: the effectiveness of RDDS is governed not by responsiveness alone, but by the ability to align chemical activation with the physical and operational constraints of clinical radiotherapy.

Within this context, the dose–response gap between radiolytic chemistry and clinically deliverable radiation doses represents the primary barrier to translation. Many systems that perform effectively under high‐dose experimental conditions fail to achieve meaningful activation under standard fractionation, underscoring a fundamental mismatch between laboratory design and clinical feasibility. Addressing this limitation requires a shift toward dose‐matched systems in which activation mechanisms are intrinsically compatible with clinically relevant irradiation, achieved through low‐energy bond design, high‐Z amplification, or direct electron‐driven processes. More fundamentally, we identify predictability as the defining requirement for clinically viable RDDS. The distinction between stochastic, microenvironment‐dependent activation and highly predictable, dose‐correlated activation provides a unifying principle for evaluating system performance. Only platforms that establish a quantitative and reproducible relationship between absorbed dose and molecular transformation can be seamlessly integrated into radiotherapy workflows. This principle reframes RDDS from responsive materials into functional components of a dosimetry‐governed therapeutic system, where drug activation can be prescribed and modulated through radiation planning.

Looking forward, the convergence of RDDS with medical physics and AI is poised to transform radiotherapy from static scheduling toward adaptive, feedback‐controlled pharmacological intervention. By integrating real‐time information on drug distribution and activation with adaptive radiation delivery, future systems will enable co‐optimization of physical dose and biochemical response. In this emerging paradigm, radiotherapy evolves into a dosimetry‐governed actuator of molecular pharmacology, where RDDS serve as transducers that convert energy deposition into controlled therapeutic output. Realizing this vision requires continued advances in mechanism‐guided design, quantitative dosimetry–chemistry coupling, and scalable manufacturing, ultimately enabling a new generation of precision oncology strategies defined by controllability, predictability, and clinical integration.

## Author Contributions

**Jianv Jiang**: conceptualization, methodology, investigation, formal analysis, writing – original draft. **Wenjing Sun**: funding acquisition, project administration, supervision, writing – review and editing. **Tanfeng Zhang**: validation, formal analysis, investigation, writing – original draft. **Junliang Dong**: project administration, funding acquisition, writing – original draft, writing – review and editing.

## Conflicts of Interest

The authors declare no conflicts of interest.

## Data Availability

Data sharing not applicable to this article as no datasets were generated or analyzed during the current study.
